# Effects of Sup35 overexpression on the formation, morphology, and physiological functions of intracellular Sup35 assemblies

**DOI:** 10.1128/aem.01703-24

**Published:** 2025-02-06

**Authors:** Jianhui Feng, Ekaterina Osmekhina, Jaakko V. I. Timonen, Markus B. Linder

**Affiliations:** 1 Department of Bioproducts and Biosystems, School of Chemical Engineering, Aalto University 3258 , Espoo, Finland; 2 The Center of Excellence in Life-Inspired Hybrid Materials (LIBER), Aalto University, Espoo, Finland; 3 Department of Applied Physics, School of Science, Aalto University, Espoo, Finland; Chalmers tekniska hogskola AB, Gothenburg, Sweden

**Keywords:** biomolecular condensates, stress response, super-resolution radial fluctuations, Sup35

## Abstract

The yeast prion protein Sup35 is aggregation-prone at high concentrations. *De novo* Sup35 prion formation occurs at a significantly increased rate after transient overexpression of Sup35 in the presence of another prion, [*PIN*^+^], but it is still a rare event. Recent studies uncovered an additional and seemingly more prevalent role of Sup35: at its physiological level, it undergoes phase separation to form reversible condensates in response to transient stress. Stress-induced reversible Sup35 condensation in the [*psi*^-^] strain enhances cellular fitness after stress ceases, whereas irreversible Sup35 aggregates in the [*PSI*^+^] strain do not confer this advantage. However, how Sup35 overexpression, which could potentially lead to irreversible aggregation, affects its condensation under stress conditions remains unclear. In this study, we used a combinatorial method to examine how different levels of Sup35 overproduction and cellular conditions affect the nature, formation, and physical properties of Sup35 assemblies in yeast cells, as well as their impacts on cellular growth. We observed notable morphological distinctions between irreversible Sup35 aggregates and reversible Sup35 condensates, possibly indicating different formation mechanisms. In addition, Sup35 aggregation caused by a very high overexpression level can strongly inhibit cell growth, diminish the formation of stress-induced condensates when Sup35 is completely aggregated, and impair cellular recovery from stress. Together, this study advances our fundamental understanding of the physical properties and formation mechanism of different Sup35 assemblies and their impacts on cellular growth. We conclude that *in vivo* studies are sensitive to overexpression and can lead to assembly routes that strongly affect functions.

**IMPORTANCE:**

The role of condensates in living cells is often studied by overexpression. For understanding their physiological role, this can be problematic. Overexpression can shift cellular functions, thereby changing the system under study, and overexpression can also affect the phase behavior of condensates by shifting the position of the system in the underlying phase diagram. Our detailed study of overexpression of Sup35 in *S. cerevisiae* shows the interplay between these factors and highlights basic features of intracellular condensation such as the balance between condensation and aggregation as well as how cellular localization and responsiveness depend on protein levels. We also apply super-resolution microscopy to highlight details within the cells.

## INTRODUCTION

The cellular environment is a dynamic milieu in which macromolecules, especially proteins, play an important role in responding to internal and external environmental cues for regulating and coordinating many cellular processes. The ability of proteins to dynamically self-assemble into diverse structures is a fundamental strategy to execute their roles on a temporal and spatial basis (1–3).

Phase separation is one of the fundamental mechanisms underlying protein self-assembly (4–6). Biological phase separation results in two phases: a condensed phase enriched with macromolecules—e.g., proteins and RNAs—and second phase, depleted in these components (4). In many biomolecular condensates, proteins containing intrinsically disordered regions (IDRs) are found abundant (7, 8). Unlike structured domains, IDRs lack a fixed tertiary structure, allowing them to adopt multiple conformations depending on the microenvironmental conditions (9). Moreover, IDRs, also referred to as low-complexity regions due to their enrichment in particular amino acids, play an important role in shaping the interactions within biomolecular condensates (9, 10). The unique properties of IDRs provide a structural and molecular basis to drive or modulate phase separation via dynamic and versatile molecular interactions with biomolecules. The interactions that hold components within biomolecule condensates strongly depend on the component concentration and environmental conditions, including pH, temperature, salt type, and salt concentration. Alteration of these parameters could influence the condensate formation and/or lead to condensates adopting different material properties (e.g., liquid-, gel- and solid-like) that dictate their cellular functionalities (11). One example is the yeast stress granule (SG), a reversible gel-like condensate formed under stress conditions by sequestering stress-responsive proteins (12–14). It serves as an organelle for a long-term and reversible storage of essential molecules to ensure cell survival.

Proteins that engage in dynamic phase separations are prone to misfolding and forming insoluble aggregates when subjected to overexpression, sequence mutation that promotes aggregation, and cellular dysfunction, leading to loss of physiological function (11, 14–16). Some of these aggregates are amyloids with a cross-β-sheet conformation, often linked to cellular toxicity and degenerative diseases (17–20). Unraveling the complexities of intracellular assemblies of a protein under different conditions is pivotal to understanding its roles in the dynamic cellular processes and how their structural conformations can be tuned. However, distinguishing between physiological condensates and insoluble aggregates in living cells based on their physical properties, which serve as straightforward indicators, is challenging due to their morphological similarities. Although their distinctions primarily lie in the material properties, techniques to examine their material properties such as fluorescence recovery after photobleaching (FRAP) are valuable but often limited by the small size of assemblies and fluorescence bleaching effects (21, 22). Therefore, a facile and systematic approach is required to explore the complexities of intracellular assemblies.

In this work, we studied the yeast protein Sup35 to understand how to evaluate effects on aggregate and condensate formation by variations in protein concentrations and cellular conditions. The protein is particularly interesting as it can alternate between different structures at different protein concentrations and cellular conditions. Sup35 is an essential multidomain protein of the translational termination complex in yeast. It consists of a C domain, a folded guanosine triphosphatase (GTPase) domain essential for its catalytic functions at its C-terminal, and a prion domain at its N-terminal (23–25). The C domain interacts with Sup45 to form a translation termination complex (23, 26). The prion domain is an IDR that can be divided into the N domain, a prion-forming region, and the M domain, which functions as a regulatory element for prion formation and stability. Overexpression of Sup35 or its prion domains (Sup35NM/N) in yeast significantly increases *de novo* formation of the [*PSI*^+^] prion, but this increase is notable only in the presence of [*PIN*^+^], the prion form of Rnq1 (27, 28). During the *de novo* generation of [*PSI*^+^] in [*PIN*^+^] cells, mature prions are observed as dot-like puncta, while filamentous intermediates are also detectable (29, 30). Various chimeric constructs of SUP35 can induce [*PSI*^+^] formation without the necessity for preexisting prion seeds. Notable examples include constructs such as Sup35N-ABeta (31) and Sup35 with oligopeptide-repeat expansions, Sup35-R2E2 (32). Recent work showed a significant role of Sup35, that is, it undergoes phase separation to form reversible condensates upon stress (33). Reversible Sup35 condensation orchestrated by the prion domain maintains the essential functionality of Sup35 and facilitates cell survival under stress conditions (33). However, conflicting evidence suggested that Sup35 condensation *in vivo* under acidic stress depends on its C-terminal domain (34). The same study also showed that Sup35 condensates are complex assemblies containing multiple cellular components, including Sup45, mRNA, and stress granule marker proteins (34).

Although significant progress has been made in understanding Sup35 condensation under stress conditions, a key question remains: how does different Sup35 concentrations impact its phase behavior, cellular growth, and stress response? The question is important because protein concentration strongly impacts IDR-mediated phase transitions. Inadequate or excessive protein concentration, compared to the saturation concentration for condensation, may result in failed protein condensation, or irreversible aggregation that might link with cellular toxicity (35). Therefore, revealing the relationship between protein concentration and phase transitions of Sup35 could presumably provide more insights into the complexity of intracellular phase transitions, which would potentially be applied to other IDRs containing proteins. Moreover, possible changes in the physical and material properties of Sup35 assemblies brought on by varying protein concentrations can also help us understand, in general, how condensates arise and are controlled *in vivo* and what morphology and dynamics condensates or aggregates acquire.

In this study, we explored a combinatorial approach to examine how alteration in protein concentrations influences the phase behavior of Sup35 in *Saccharomyces cerevisiae*. Our approach to investigate intracellular Sup35 assemblies encompassing both *in vivo* analysis, such as fluorescence microscopy (FM), flow cytometry, and super-resolution image processing, and *in vitro* biochemical assays. The *in vivo* analysis serves to examine the physical properties and reversibility (as a proxy for material properties) of intracellular assemblies, while the biochemical assays are conducted to complement and validate the findings obtained *in vivo*. Here, following the proposed workflow, we show the stress-induced Sup35 condensates are smaller in size, rounder in shape, and smoother in periphery than Sup35 irreversible aggregates that are typically bigger and often irregular. Notably, Sup35 under mild overexpression levels exhibits nearly unaffected reversible stress-induced condensation, while a very high overexpression of Sup35 elicits significant Sup35 aggregation, which diminishes the formation of stress-induced condensates in cells containing fully aggregated Sup35. Additionally, Sup35 aggregation is found to be associated with cellular growth inhibition.

## RESULTS

### A tunable expression system allows accurate control of protein expression levels in yeast

**Fig 1:**
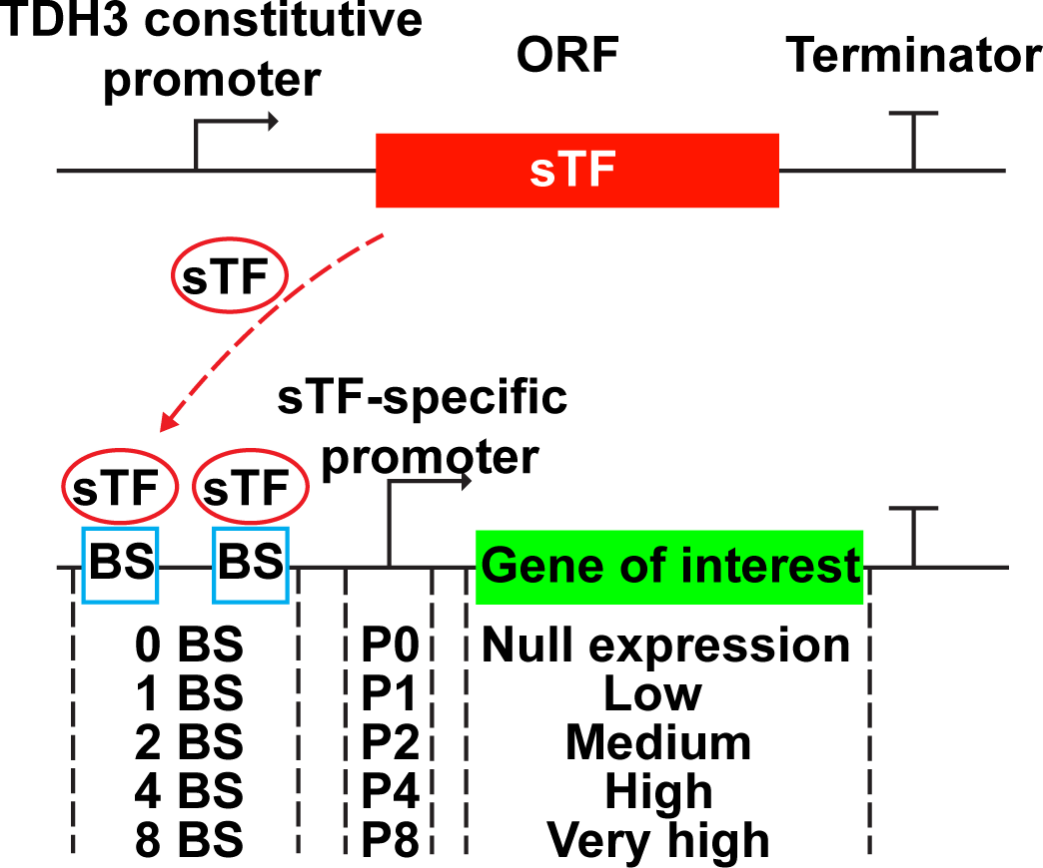
Overexpression of Sup35 at different levels using a robust protein expression system (SynPro) in *S. cerevisiae*. The expression cassette of the synthetic transcriptional factor (sTF) is integrated into the yeast’s genome, and its expression is controlled by the TDH3 promoter, ensuring a constitutive and low expression of sTF. The expression cassette of the gene of interest is introduced into yeast cells with an episomal plasmid, and its expression is controlled by an sTF-specific promoter that was engineered to contain different number of binding sites (BS) of sTF. The binding between sTF and BS activates the expression of the protein of interest, and the number of BS is proportional to the expression level. The promoters contain five different numbers of binding sites (BS): zero, one, two, four, and eight, referred to as P0, P1, P2, P4, and P8, respectively. These correspond to null, low, medium, high, and very high levels of protein expression.

To regulate intracellular Sup35 concentration, we chose an expression system (SynPro) developed by Rantasalo et al., which allows accurate control of gene expression in *Saccharomyces cerevisiae* (36). In SynPro, the expression level increases with the number of binding sites of a synthetic transcriptional factor (sTF), which were engineered upstream of the sTF-dependent promoter (Fig. 1). The promoters were modified to contain zero, one, two, four, or eight binding sites (denoted as P0, P1, P2, P4, or P8, respectively). SynPro can provide varying protein expression levels that correlate positively with promoter strength, ranging from null (i.e., no expression) to low, moderate, high, or very high expression levels. This was demonstrated by using SynPro to express different levels of a highly soluble fluorescent protein, Venus (36).

**Fig 2:**
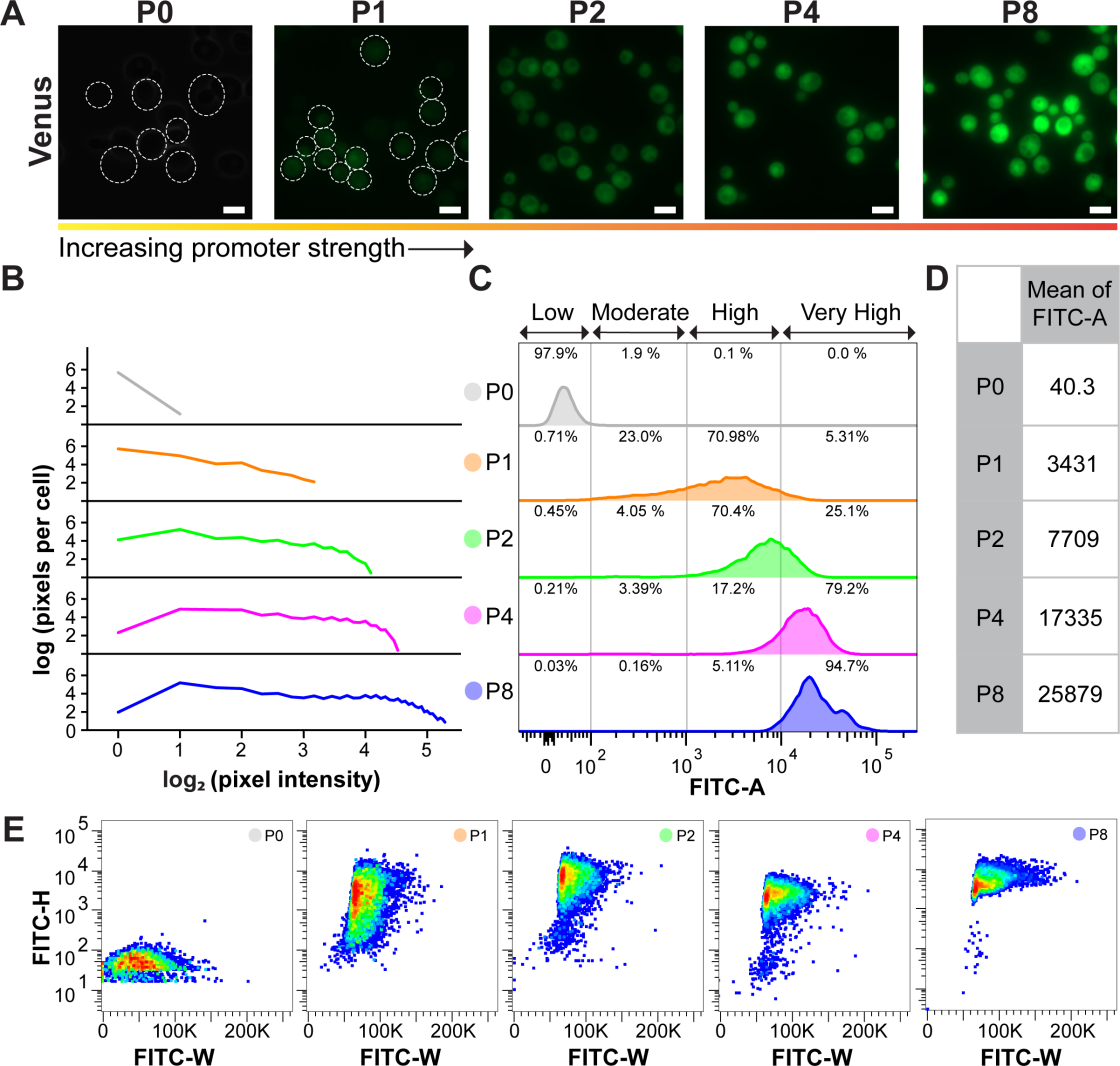
Validation of the protein expression system (SynPro) developed in *S. cerevisiae* with a reporter protein, Venus. (**A**) Fluorescence micrographs of *S. cerevisiae* expressing Venus under different promoter strengths. All images were acquired with identical settings so that the fluorescence intensity of Venus in images can be compared between cells expressing Venus under different promoter strengths. Dashed lines indicate the cell border. Scale bar: 5 µm. (**B**) Pixel distribution per cell at different pixel intensity levels for cells with different levels of Venus. Around 20 microscopy images containing around 30 cells of each sample were used for quantification. The lines represent the mean of the number of pixels per cells at different pixel intensity levels. (**C**) Flow cytometry analysis on cell cultures producing Venus at different promoter strengths. Four subpopulations were divided based on the fluorescence intensity (FITC-A) of Venus: Low FITC-A, Moderate FITC-A, High FITC-A, and Very High FITC-A. The number shown in percentage represents the percentage of cells in the four subpopulations. (**D**) A table of the mean FITC-A for each cell culture producing Venus under different promoter strengths. (**E**) FITC-H versus FITC-W scatter plots of each cell culture producing Venus. All flow cytometry data and scatter plots were derived from 10,000 events.

To confirm the functionality and acquire a better understanding of SynPro, we expressed Venus with SynPro and used fluorescence microscopy, immunoblotting, and flow cytometry to analyze its expression level. A clear increase in both fluorescence intensity and the number of higher-intensity pixels was observed in the Venus-expressing cells, in response to the increasing promoter strength (Fig. 2A and B). Immunoblotting with densitometric measurement showed that the yield of Venus increased with promoter strength ( Fig. S1A and B; Table S1). Consistently, flow cytometry data also revealed that a stronger promoter resulted in an increased number of cells homogenously shifting to a higher FITC-A range (i.e., higher fluorescence), along with an increased mean FITC-A (Fig. 2C and D). While P8-Venus cells exhibited the highest mean FITC-A, a nearly proportionate increase in mean FITC-A was only observed from P1- to P4-Venus cells (Fig. 2D). It is worth mentioning that the promoter P0 yielded a negligible average FITC-A, and nearly 98% of cell populations located in a range of FITC-A classified as “low” (< 10^2^ arbitrary unit) (Fig. 2C). Thus, cells with fluorescence within this “low” range were considered as nonfluorescence-producing cells, which was applied to the following Sup35 analysis. Taken together, SynPro provides a robust and accurate control on gene expression proportional to the promoter strength.

Recent studies showed that the scatter plot of the height (FITC-H) versus width (FITC-W) of fluorescence can be used to distinguish between a soluble and aggregated form of a protein in yeast cells (37, 38). The FITC-A is defined by these two values measured by flow cytometry. Should the fluorescence within a cell be uniformly distributed, a pattern with a narrow FITC-H and a wide FITC-W is generated, in comparison to a wide FITC-H and narrow FITC-W pattern generated from a cell with puncta (37). FM images showed Venus consistently exhibited diffuse fluorescence and did not form puncta in cells, irrespective of the promoter strength (Fig. 2A). As expected, all Venus-producing cells displayed a pattern with a narrow FITC-H and wide FITC-W (Fig. 2E). Such a pattern was more pronounced, with the FITC-W range becoming broader, when Venus was overexpressed at higher levels, indicating that fluorescence remained dispersed in cells (Fig. 2E). As the scatter plot of FITC-H versus FITC-W showed an anticipated pattern for soluble protein, this could become an analytical method to distinguish different states of Sup35.

### High overexpression levels of Sup35 lead to the formation of non-prion Sup35 aggregates

**Fig 3:**
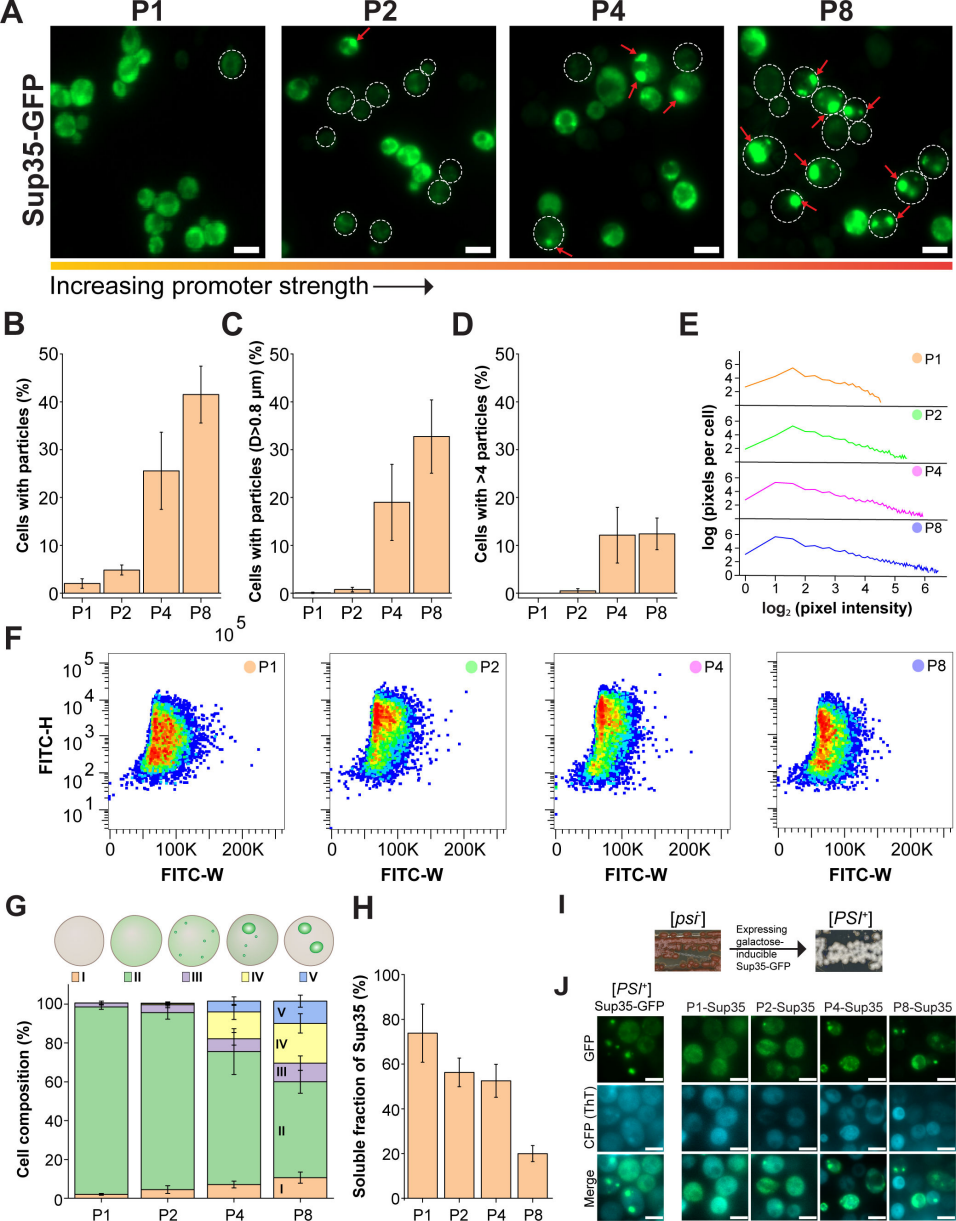
High overexpression levels of Sup35 lead to the formation of non-prion Sup35 aggregates. (**A**) Fluorescence micrographs of *S. cerevisiae* expressing Sup35-GFP under different promoter strengths. Images were taken at identical settings but processed with different parameters for better visualization of the Sup35 assemblies. Thus, the fluorescence intensity of Sup35-GFP shown in images is not comparable. Red arrows indicate the aggregate-like Sup35 particles. Dashed lines indicate the cell border. (**B**) Percentages of cells with fluorescent particles, (**C**) percentages of cells with fluorescent particles large than 0.8 µm in diameter (**D**), and (**D**) proportions of cells with more than four fluorescent particles per cell in cultures with different overexpression levels of Sup35. (**E**) Pixel distribution per cell at different pixel intensity levels for cells overexpressing different levels of Sup35. Around 20 microscopy images containing around 30 cells of each sample were used for quantification. (**F**) FITC-H versus FITC-W scatter plots of each cell culture producing Sup35-GFP. All flow cytometry data and scatter plots were derived from 10,000 events. (**G**) Percentage of cells with a specific fluorescent pattern in cells overproducing Sup35. (i) Cells with low or no detectable diffuse fluorescence, (ii) cells with only diffuse fluorescence, (iii) cells containing only small particles (less than 0.8 µm in diameter), (iv) cells with both large particles (over 0.8 µm in diameter) and a few small particles and/or diffuse fluorescence, and (v) cells with large particles and low or nondetectable fluorescence. (**H**) Percentages of the soluble fraction of Sup35 in cells overproducing Sup35. (**I**) Inducing the [psi^–^] yeast strain to [PSI^+^] by expressing a galactose-inducible Sup35-GFP in a [psi^–^ PIN^+^] strain on a YPD plate. To determine the Sup35 prion formation, a simple phenotypic assay was used. The prion formation mediates the read-through of the stop codon, leading to colony color change from red to white on a YPD plate. Scale bar of images of yeast colonies: 1 cm. (**J**) Thioflavin-T (ThT) staining for Sup35-GFP at different overexpression levels. The ThT fluorescence was visualized in the CFP channel. Scale bars of all microscopy images are 5 µm. For all plots, error bars show the standard deviations.

Sup35 was tagged at its C-terminus with a fluorescent protein GFP, and the resulting fusion protein, Sup35-GFP, was overexpressed with SynPro in *S. cerevisiae* cells. Hereafter, we omit GFP when referring to Sup35-GFP to reduce complexity. Sup35 under varying overexpression levels produced a diversity of fluorescent patterns in cells (Fig. 3A). Under the lowest overexpression level P1, Sup35 predominantly exhibited dispersed fluorescence, with a nearly negligible number (2%) of cells containing fluorescent particles (Fig. 3B). Similarly, only a low percentage of cells (5%) contained a few round fluorescent particles upon overexpressing Sup35 at a moderate level (P2), whereas most cells still exhibited diffuse fluorescence (Fig. 3B). In addition, most of these round particles in P1- and P2-Sup35 cells were small, measuring less than 0.8 µm in diameter (Fig. 3C; Fig. S2A). Particles over 0.8 µm in diameter were considered large, while those under 0.8 µm were considered small. These collectively suggested that Sup35 molecules remained primarily in a monomeric or oligomeric state upon low or moderate overexpression by SynPro, and the critical concentration required for Sup35 nucleation was not reached. Further elevating the overexpression level of Sup35 to P4 and P8 increased the percentage of cells containing particles, the formation of large particles (over 0.8 µm in diameter), and the number of particles per cells (Fig. 3B through D ; Table S3). P8-Sup35 cells showed a more pronounced increase than P4-Sup35 cells in both the percentage of particle-containing cells and the proportion of cells with large particles (Fig. 3B and C). We then applied the same methods used for Venus to study Sup35. Quantitative image analysis on FM images showed that from P1 to P8, there was a clear and gradual elevation in the number of higher-intensity pixels (Fig. 3E), indicating that increasing protein expression levels led to greater accumulation of proteins into large agglomerates. This tendency was also reflected in the FITC-H versus FITC-W scatter plot from flow cytometry experiments (Fig. 3F), where the FITC-W range became slightly narrower as the Sup35 expression levels increased, implying fluorescence became less dispersed (i.e., more proteins were concentrated into fluorescent puncta).

Closer examination on FM images revealed varying fluorescence patterns in cells overproducing Sup35 (Fig. 3G), categorized as follows: (i) cells with low or nondetectable diffuse fluorescence, (ii) cells with only diffuse fluorescence, (iii) cells containing only small particles (less than 0.8 µm in diameter), (iv) cells with both large particles (over 0.8 µm in diameter) and a few small particles and/or diffuse fluorescence, and (v) cells with large particles and low or nondetectable fluorescence. Higher overexpression levels of Sup35 led to a transition from soluble protein in most cells to small particles and then to larger particles in an increasing number of cells (Fig. 3G; Table S4). Time-lapse imaging further illustrated this progression over time at a single-cell level in P8-Sup35 cells (Fig. S3). Moreover, some of these large particles observed in P4- and P8-cells were irregular, in contrast to the round shape and small size adopted by Sup35 condensates (33, 34). Together with the aggregation-prone nature of Sup35, these results collectively led us to suspect that the Sup35 particles formed under high overexpression levels were insoluble aggregates, possibly formed due to the anisotropic intramolecular interactions between Sup35 molecules. To evaluate this proposal, we compared the expression levels and the solubility of Sup35 at different promoter strengths with immunoblotting, followed by densitometry. Expression of Sup35 driven by SynPro resulted in approximately fivefold to 15-fold higher levels, depending on the promoter strength, compared to Sup35-GFP expressed in a derivative of the [*psi*^–^ *PIN*^+^] YJW584 strain, YJW584-ΔSup35 (Fig. S1C and D; Table S1). The derivative strain features a deletion of the chromosomal Sup35 gene, with Sup35-GFP expressed from a centromeric plasmid under the control of the Sup35 endogenous promoter. Thus, the expression level of Sup35-GFP in the derivative strain can be interpreted as the physiological level of Sup35. Furthermore, it was found that the soluble fraction of Sup35 decreased with the increase in promoter strength, with P8-Sup35 showing the lowest soluble fraction at around 20% (Fig. 3H; Table S2), indicating P8-Sup35 cells produced a significant amount of insoluble forms of Sup35. Therefore, we refer here and below to these Sup35 particles in cells overproducing Sup35 as aggregates. However, we note that this classification is based primarily on their morphological properties observed through fluorescence microscopy and protein solubility.

To investigate whether the Sup35 aggregates induced by the SynPro system are prion aggregates, we stained the cells with thioflavin T (ThT), a dye that specifically binds to amyloid-like fibrillar aggregates (39, 40). The dye was shown to bind Sup35 prion aggregates that contain amyloid-like structures (41–43). To develop a positive control for this characterization, we expressed Sup35-GFP with a galactose-inducible plasmid in the [*psi*^–^ *PIN*^+^] YJW584 strain (33, 44). The strain contains a premature stop codon in the *ADE1* gene, enabling a straightforward phenotypic assay to assess [*PSI*^+^] formation. In this assay, prion-mediated read-through of the stop codon induces a colony color change from red to white on a YPD plate (45). White colonies were spotted and inoculated for growth and later fluorescence microscopy examination (Fig. 3I). Sup35 foci stained positively with ThT, suggesting they were prions, consistent with previous findings (Fig. 3J, left column) (42, 43). In contrast, aggregates formed by P2-, P4-, and P8-Sup35 cells did not show ThT positivity in the same assay (Fig. 3J). P8-Sup35 was further characterized with another amyloid-specific dye Amytracker, which also showed negative staining (Fig. S4), reinforcing the indication that Sup35 aggregates formed in the SynPro system lack amyloid-like structures. To assess whether Sup35 aggregation in our system is influenced by other preexisting prions, such as [*PIN*^+^], the yeast strains of SynPro were cured of potentially all prions by growing on YPD in the presence of 5 mM guanidine HCl (GuHCl) for three passes, followed by streaking the colonies on YPD plates (45–47). Sup35 at different overexpression levels showed indistinguishable behaviors in cured and noncured cells (Fig. 3A; Fig. S5). This suggested that either the noncured cells themselves lack prions that interfere with Sup35 aggregation or that Sup35 aggregation in the SynPro system occurs independently of other prions, if such prions are present in our noncured yeast strains. Hence, the properties of Sup35 aggregates formed in the SynPro system can be interpreted as the non-amyloid Sup35 aggregates formed in a [*psi^–^pin*^–^] strain.

### Sup35 aggregates are associated with cellular growth inhibition

**Fig 4:**
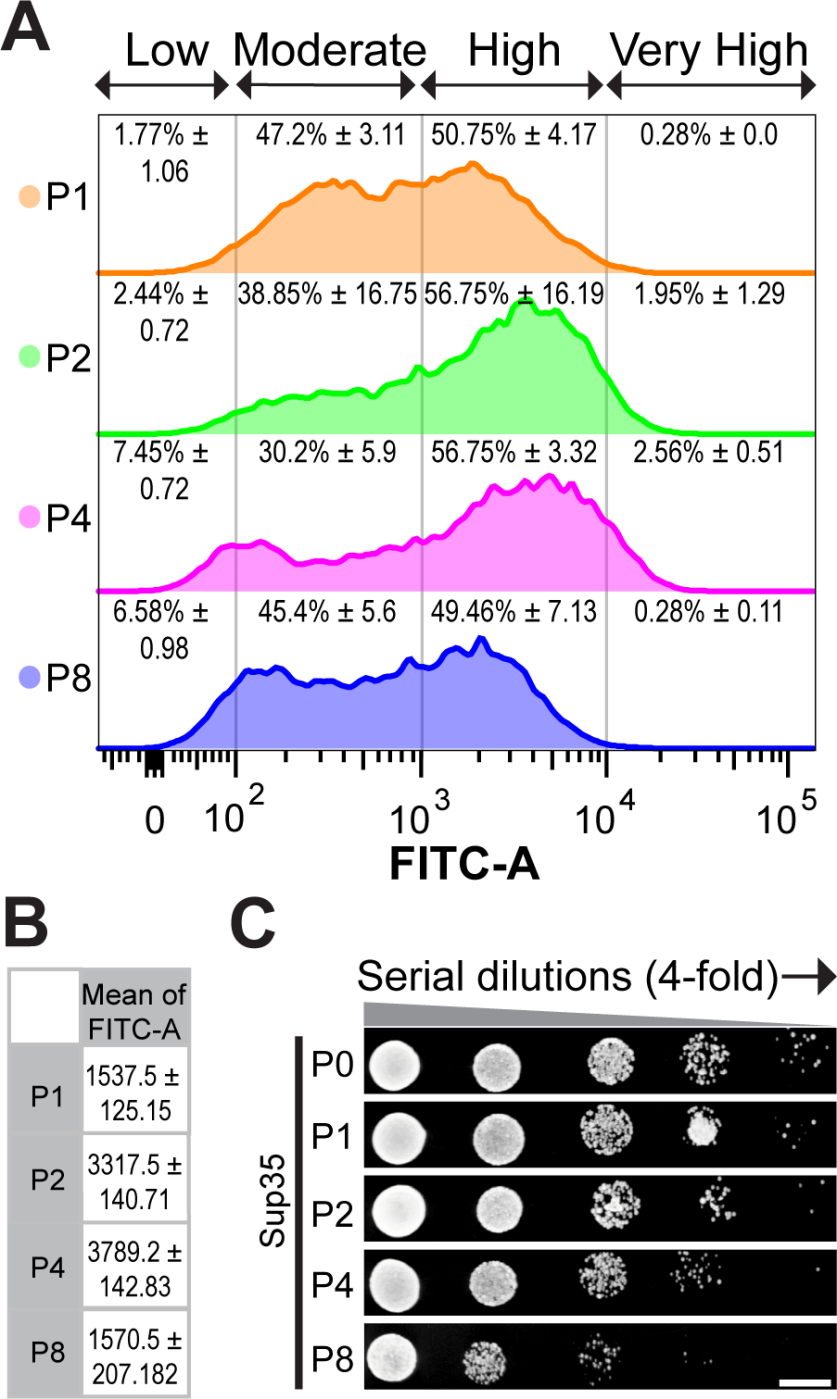
Sup35 aggregates are associated with cellular growth inhibition. (**A**) Flow cytometry analysis on cell cultures producing Sup35 at different overexpression levels. Four subpopulations were divided based on the fluorescence intensity (FITC-A) of GFP: Low FITC-A, Moderate FITC-A, High FITC-A, and Very High FITC-A. The number shown in percentage represents the percentage of cells in the four subpopulations, along with their corresponding standard deviations. (**B**) A table of mean FITC-A for each cell culture overproducing Sup35. (**C**) Spot-titer growth assay of yeast cells grown in the same SD-Leu-Ura agar plate with different overexpression levels of Sup35 under the identical condition with the same starting OD_600_. Scale bar: 1 cm.

Analysis of FM images showed the number of cells with nondetectable fluorescence increased with promoter strength (Fig. 3A and G). A similar trend was also observed from the flow cytometry data (cells with FITC-A <10^2^)were considered lacking detectable fluorescence) (Fig. 4A). For instance, cells expressing Sup35 at the highest level (P8) surprisingly displayed FITC-A as low as that of P1-Sup35 cells, although the flow cytometry data revealed an increase in both the number of cells producing stronger fluorescence and mean FITC-A from P1- to P4-Sup35 cells (Fig. 4A and B). There was also a significant increase in the subpopulation of cells with low fluorescence intensity (FITC <10^2^)as Sup35 levels increased (Fig. 4A). The proportion of cells with combined low and moderate fluorescence (i.e., FITC <10^3^)in P8-Sup35 was comparable to that of P1-Sup35 (Fig. 4A). Moreover, despite having the highest promoter strength, P8-Sup35 cells exhibited a lower protein yield than those of P2- and P4-Sup35 cells. However, the protein yield of Venus increased with the promoter strength (Fig. S1C and D; Table S1). This likely indicated that overexpression of Sup35 at a very high level (i.e., P8) might impose protein production burden to the cells.

Furthermore, a higher expression of Sup35 was correlated with increased cellular growth inhibition, with P4-Sup35 and P8-Sup35 cells exhibiting the most pronounced effects (Fig. 4C). In contrast, no significant difference in growth was observed in cells producing different levels of Venus (Fig. S6A). These results together suggested excessive Sup35 levels led to Sup35 aggregation, which may be toxic to cells, resulting in cell death or impaired growth and reduced production of Sup35. Consistently, studies also showed that Sup35 belongs to the class of dosage-sensitive yeast proteins capable of inducing cellular toxicity when its concentration exceeds a certain threshold (35, 48).

### Stress-induced Sup35 condensation can be interfered by Sup35 aggregation before stress exposure

**Fig 5:**
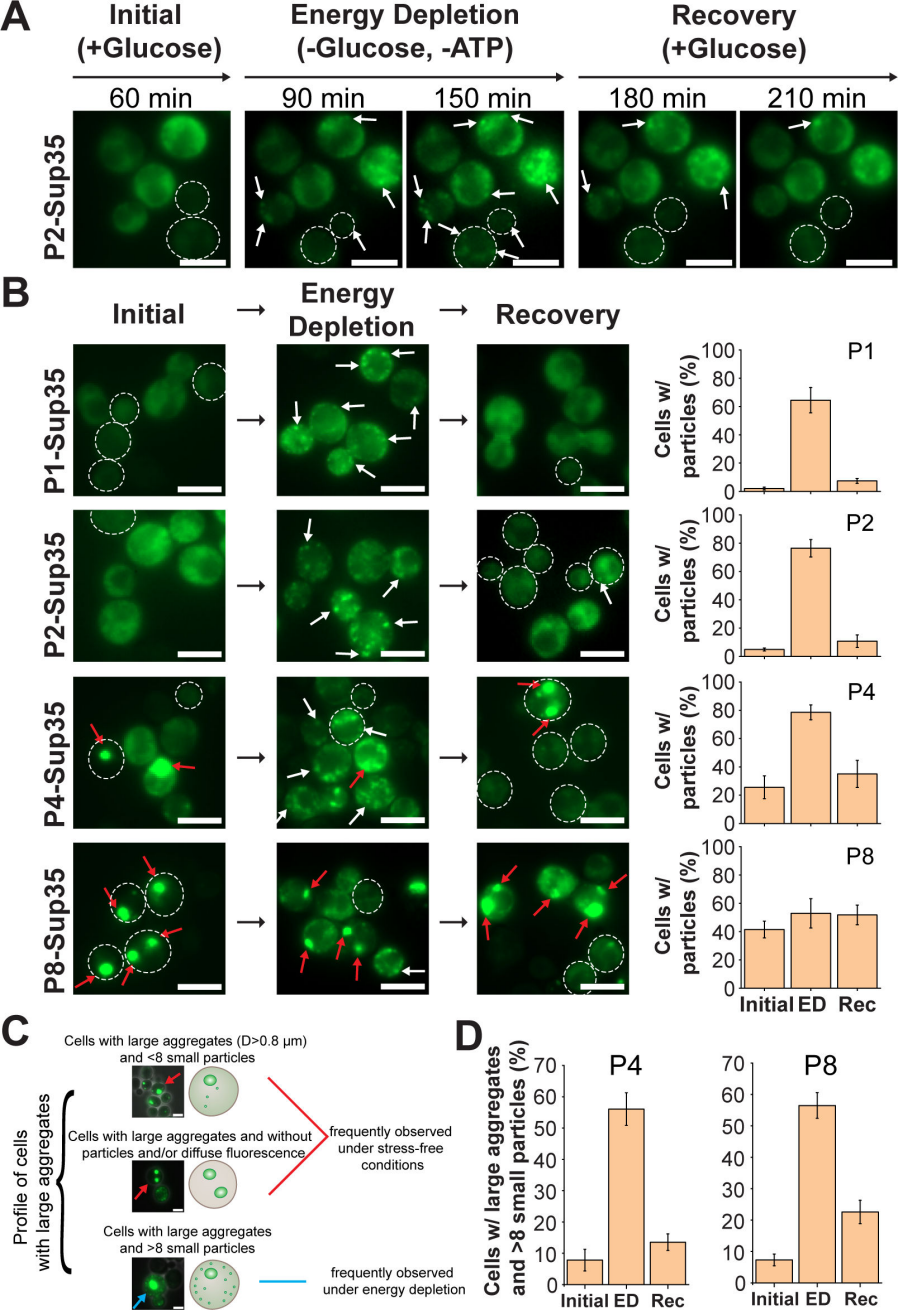
The effect of different overexpression levels of Sup35 on stress-induced Sup35 condensation. (**A**) Fluorescence micrographs taken from a time-lapse experiment of *S. cerevisiae* expressing P2-Sup35. Exponentially growing cells were transferred to a microfluidic device and grown initially in fresh media for 60 minutes prior to energy depletion. Subsequently, cells were recovered with the addition of growth media after 90 minutes of energy depletion. White arrows indicate condensate-like structures. (**B**) Fluorescence micrographs of *S. cerevisiae* expressing Sup35-GFP under different promoter strengths during exponential growth, during energy depletion, and after resupplying cells with fresh media. White arrows indicate condensate-like structures. Red arrows indicate aggregate-like structures. All images were acquired at an identical setting but processed with different parameters for better visualization purposes. Graph representation of the quantification of cells containing particles. Around 20 fluorescence microscopy images containing around 30 cells of each sample under different conditions were used for quantification. Scale bars of all fluorescence microscopy images are 5 µm. (**C**) Scheme of the profile of cells with large aggregates in cells producing P4- and P8-Sup35. (**D**) Proportions of cells containing large aggregates (D > 0.8 µm) and >8 small particles under different cultivation conditions in cells producing P4- (left) and P8-Sup35 (right). For all plots, error bars show standard deviations (SDs).

**Fig 6:**
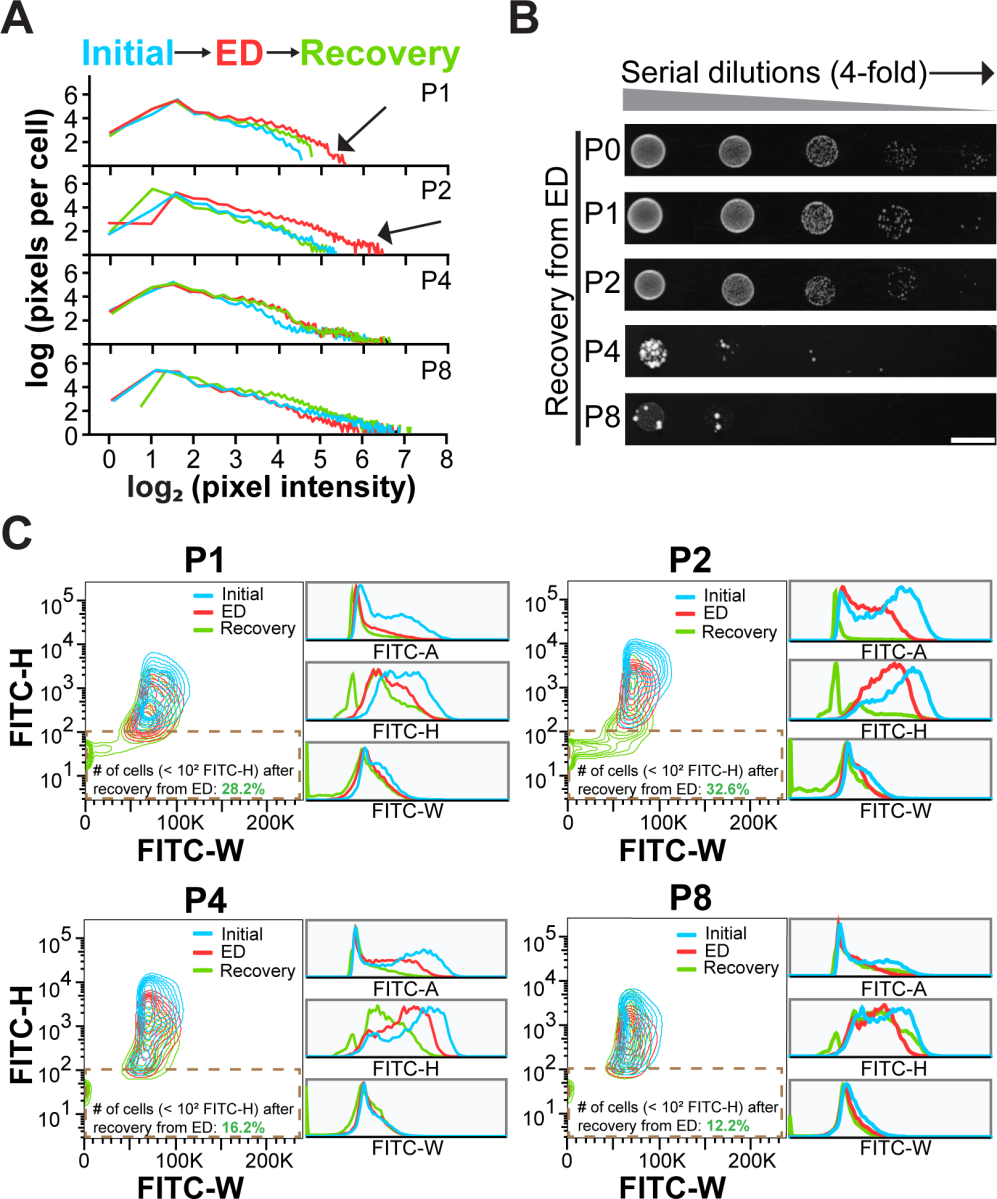
The effect of different overexpression levels of Sup35 on stress-induced Sup35 condensation and cellular stress response. (**A**) Pixel distribution per cell at different pixel intensity levels for cells with different levels of Sup35-GFP under different conditions. The blue, red, and green lines represent the mean of the number pixels per cell at different pixel intensity levels under initial condition, under stress condition, and after recovery from stress, respectively. The black arrows indicate the increase in the number of higher-intensity pixels under stress conditions compared to other conditions. (**B**) Spot-titer growth assay of cells with different overexpression levels of Sup35 that were recovered by resupplying cells with fresh media SD-Leu-Ura after energy depletion. The corresponding control plate with the same cultures before stress is shown in Fig. 4C. Scale bar: 1 cm. (**C**) FITC-H versus FITC-W contour plots of each cell culture producing Sup35-GFP under different conditions. Histograms are the overlay of FITC-A, FITC-H, or FITC-W of Sup35-GFP cells under different conditions. Dashed brown boxes represent the subpopulation of cells with an FITC-H below 10^2^ arbitrary units that are classified as non-fluorescent cells after recovery from stress. All flow cytometry data and plots were derived from 10,000 events.

Sup35 can assemble into reversible condensates under stress conditions, e.g., energy depletion (33, 34, 49). Here, we investigated how different Sup35 concentrations, leading to different assembled states of Sup35 prior to stress, affect its reversible condensation upon stress. Under low (P1) or moderate (P2) overexpression levels, in which Sup35 was primarily soluble before exposure to stress, as previously shown, it assembled into various small particles around the cytoplasm after depleting the yeast cells of energy (Fig. 5A and B; Table S5). Those particles shared a high similarity in physical properties to the intracellular Sup35 condensates in the study of Franzmann et al (33). For example, both are small (D < 0.8 µm), round, and distributed around the cytoplasm in a similar manner (Fig. S2B). Therefore, these small particles were initially interpreted as Sup35 condensates. Notably, upon removal of stress and subsequently resupplying energy sources to the cells, a majority of Sup35 condensates disappeared after 2 hours (Fig. 5A and B). Concomitantly, most cells were depleted of condensates and exhibited evenly distributed fluorescence again (Fig. 5B), confirming these stress-induced Sup35 condensates were reversible, as anticipated. The reversibility of Sup35 condensates in P1- and P2-Sup35 cells was also reflected in the pixel intensity analysis, which showed the number of higher-intensity pixels increased upon energy depletion but almost retreated to their initial levels after supplying yeast with energy sources (Fig. 6A).

P4-Sup35, which caused both less Sup35 aggregation and cellular growth defect compared to P8-Sup35 as shown previously (Fig. 4A through C), still underwent stress-induced reversible condensation. This was demonstrated by the formation of many small condensates upon exposure to stress in an increasing number of P4-Sup35 cells, followed by their disappearance from most cells upon energy restoration (Fig. 5B). For P8-Sup35, the number of cells with particles did not change significantly before, during, and after stress (Fig. 5B), although many small particles morphologically similar to condensates were observed in some stressed cells.

Some interesting observations were made: (i) in both P4- and P8-Sup35 cells, preexisting large aggregates were stable, unaffected by stress or energy recovery (Fig. 5B, indicated by red arrows). Their irreversibility and insensitivity to stress, thus, represented the critical features different from the reversible Sup35 condensates. (ii) stress-induced condensates primarily appeared in cells lacking large aggregates (Fig. 5B, indicated by white arrows). (iii) cells with large aggregates, especially in the case of P8-Sup35, can be found containing many small particles morphologically similar to condensates under stress conditions (Fig. S7A), although less frequently. These observations likely suggested that condensate formation might not be necessarily suppressed in the cells containing aggregates, but it might be diminished when Sup35 forms aggregates.

To further evaluate whether pre-existing Sup35 aggregation would affect its condensation during stress, we need to separate small condensates from Sup35 aggregates, which was not achieved in the above analysis (Fig. 5B). However, P4- and P8-Sup35 can form aggregates resembling the size and shape of stress-induced condensates, making them impossible to distinguish by physical properties. Nonetheless, we noted that condensates appeared in a cell with higher abundance, with usually over eight of them per cell (see P1- and P2-Sup35 under stress conditions in Fig. 5B). This was contrasted with Sup35 aggregates typically observed in P4- and P8-Sup35 cells, where the number of them per cell is around five or fewer in most cases (Fig. 5C). Additionally, the formation of large aggregates with a diameter larger than 0.8 *µ*m is driven by high Sup35 concentration rather than stress. That said, if a cell contains both large aggregates and more than eight small particles during stress conditions, this can be interpreted that Sup35 condensation occurs in that cell even with the presence of aggregates. Thus, we focused only on P4- and P8-Sup35 cells with large aggregates over 0.8 µm in diameter and counted the number of such cells containing more than eight particles to determine whether pre-aggregated Sup35 influences its condensation (Fig. 5C). Cells containing both large aggregates and over eight smaller particles were rare before stress exposure in both cells, representing only 10% of total cells containing large aggregates, but this proportion increased to approximately 50% under stress (Fig. 5D; Table S6). After energy restoration, this decreased to around 20% in P4-Sup35 and 30% in P8-Sup35 cells (Fig. 5D; Table S6). These results suggested that irreversible aggregation of Sup35 did not necessarily affect its condensation under stress conditions. However, in the extreme case, where cells only contained large aggregates without detectable fluorescence, Sup35 condensates were not formed under stress condition ( Fig. S7B). Although this analysis could not definitively determine whether preexisting aggregates impact Sup35 condensation, it seems that Sup35 condensation likely depends on the availability of soluble Sup35 rather than Sup35 aggregation *per se*. The suppression of Sup35 condensation might become notable only when Sup35 is fully aggregated in a cell.

We wondered whether the scatter plot of FITC-H versus FITC-W from flow cytometry can distinguish different states of Sup35 under different conditions. During stress, an obvious decrease was observed in both FITC-A and the range of FITC-H across all promoter strengths, in comparison to their levels before stress (Fig. 6C; Fig. S8). This phenomenon is likely because the decrease in cytosolic pH to the acidic value in energy-depleted cells causes fluorescence quenching. The FITC-W range was anticipated to become narrower due to stress-induced condensate formation, but it did not exhibit such a trend irrespective of the overexpression levels (Fig. 6C; Fig. S8). Similarly, the disassembly of Sup35 condensates after recovery from stress did not induce an obvious change in FITC-W (when the FITC-H is above the threshold of 10^2^ arbitrary units) (Fig. 6C). These results together suggested the flow cytometry may not be as sensitive as fluorescence microscopy in detecting the subtle changes in the fluorescence pattern formed by Sup35.

On the other hand, the plots unexpectedly revealed that a substantial number of nonfluorescent cells (FITC-H <10^2^) emerged after recovery from stress across all overexpression levels of Sup35, in comparison to the negligible amount of such cells under the initial condition and energy depletion (Fig. 6C). More specifically, moderate overexpression levels (e.g., P1 and P2), which gave rise to relatively lower Sup35 aggregate load under initial condition, generated a higher proportion of nonfluorescent cells compared to the cells with high Sup35 overexpression levels (e.g., P4 and P8). The latter exhibited a considerable aggregate load in cells. The nonfluorescent cells were likely the newly budded yeast cells after energy restoration, which had not yet produced sufficient number of fluorescent proteins. This assumption was based on the fact that cells with lower aggregate load can restore cellular growth from stress more rapidly and effectively than cells with a higher aggregate load (50, 51). Spot-titer growth assay corroborated this hypothesis, showing that P4- and P8-Sup35 cells exhibited a reduced fitness when recovering from stress, whereas P0-, P1-, P2-Sup35 cells, and cells producing Venus did not show such a decline (Fig. 6B; Fig. S6B). However, since cells with a higher load of aggregates exhibited greater growth inhibition even prior to stress exposure, it remains unclear whether the impact on cellular fitness during stress recovery is due to the substantial overproduction of Sup35 or the formation of stress induced condensates.

### Super-resolution imaging technique has a potential to distinguish Sup35 at different states

The physical properties of small intracellular structures in yeast cells are challenging to be resolved with standard fluorescence microscopy due to the diffraction limit. We wondered whether a super-resolution imaging technique could help visually distinguish different states of Sup35 based on their physical properties, particularly in the case of condensates and irreversible aggregates. Super-resolution radial fluctuation (SRRF), a computational technique that enhances the resolution of fluorescence microscopy images by analyzing the fluctuations in the intensity of fluorophores, was thus applied to acquire super-resolution images of Sup35 in yeasts (52, 53).

**Fig 7:**
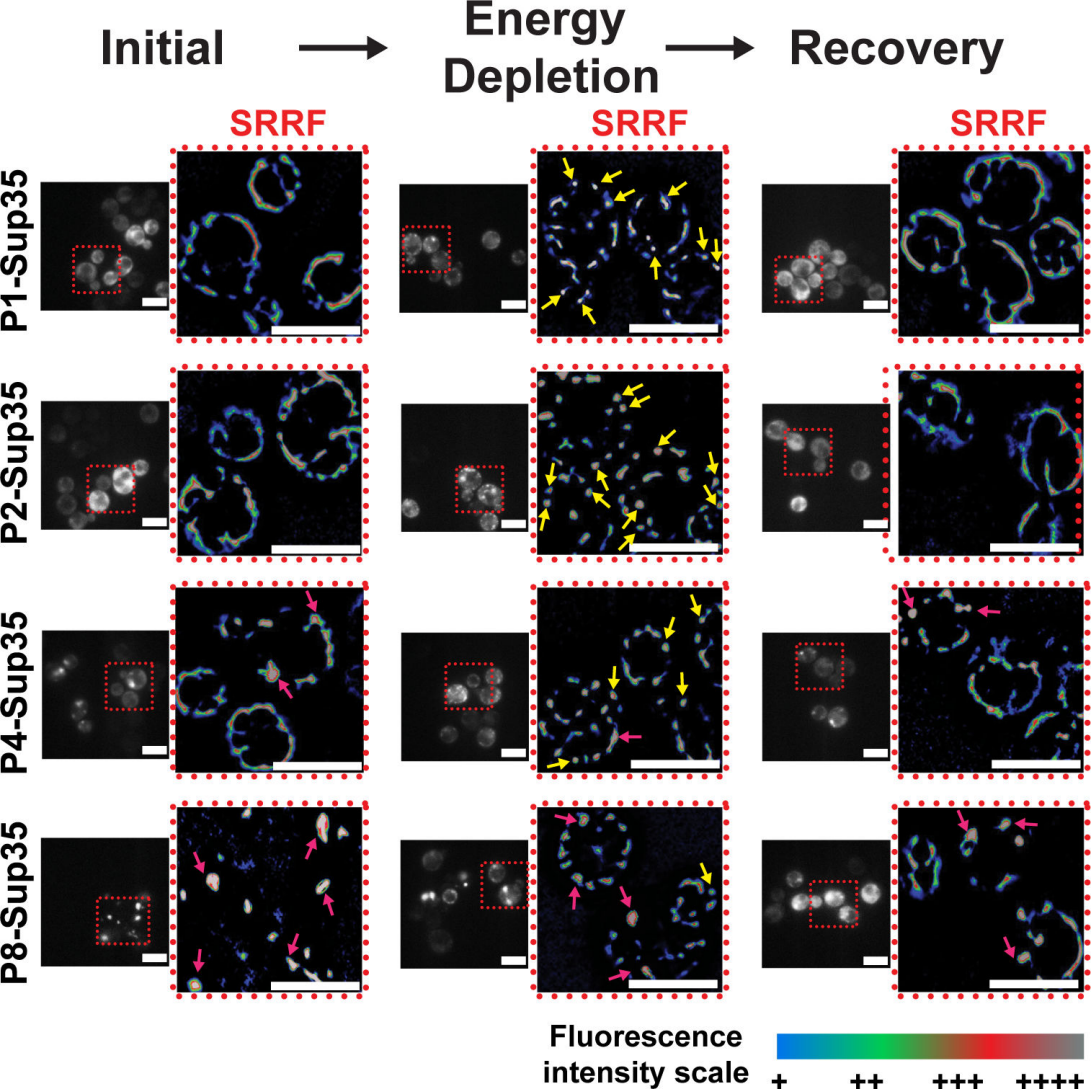
Super-resolution radial fluctuation (SRRF) analysis of Sup35 of varying overexpression levels in different conditions. SRRF images circled by a red dashed box represent the magnified region (marked with a red dashed box) of the standard fluorescence microscopy images. The fluorescence intensity of the fluorophore in SRRF images was pseudo-color-coded, with gray representing the highest intensity, followed by red, green, and blue representing intensity in decreasing order. Yellow arrows point to condensate-like particles, while magenta arrows point to aggregate-like particles. All scale bars are 5 µm.

The SRRF images of cells producing Sup35 at low (P1) or medium (P2) expression levels under normal conditions, where most Sup35 remained soluble, revealed that Sup35 displayed a dispersed fluorescence pattern around the cellular periphery and formed just a few puncta. (Fig. 7; Fig. S9). In contrast, when subjected to energy depletion, the super-resolved structures of Sup35 condensates displayed as multiple round particles individually residing around cells. Such particles had smooth edges. The recovery of cells from stress, which should result in the dissolution of most Sup35 condensates, gave rise to an Sup35 pattern in SRRF images, highly similar to that observed in cells prior to stress. These observations suggested, consistent with fluorescence microscopy, that SRRF can reveal the reversible stress-induced formation of Sup35 condensates, which are distinct from the soluble Sup35 under stress-free conditions.

The Sup35 aggregates widely present in cells with a high (P4) or very high (P8) overproduction of Sup35 under initial conditions are irregularly shaped with a rough edge, as in the SRRF images, in stark contrast to the smaller, rounder, and smoother stress-induced Sup35 condensates at lower concentrations, as mentioned above (Fig. 7). In P4-Sup35 or P8-Sup35 cells, we also observed the formation of the particles with physical properties similar to those of P1- or P2-Sup35 condensates under stress conditions, corroborating our previous finding that, at high concentration, a fraction of Sup35 can still form stress-induced condensates. However, there were some small particles already in these cells under the initial condition. These small particles could be just a consequence of partial aggregation of Sup35, but SRRF images revealed they had a similar appearance to that of the stress-induced condensates. The inability of SRRF to differentiate the small Sup35 aggregates from the stress-induced Sup35 condensates could potentially complicate the interpretation of the reversibility of Sup35 condensates when Sup35 was highly overexpressed. That is because, by just examining the SRRF images, it was still uncertain whether the small particles observed in the recovery state of cells with high Sup35 expression were stress-induced condensates or irreversible Sup35 aggregates.

Other than the limitation to distinguish cellular structures that are physically similar but differ in reversibility, such as protein condensates versus irreversible aggregates, another caveat is SRRF is an algorithm-based post-processing method that might contribute to the formation of image artifacts, leading to misinterpretation of structural formation. Therefore, SSRF should couple with other characterization methods to correctly discern physical–chemical properties of protein assemblies in cells.

## DISCUSSION

Previous data have shown that overexpression of the complete Sup35 protein or Sup35 derivatives (Sup35N or Sup35NM) in [*PIN*^+^] strain can cause *de novo* formation of Sup35 prion (27). The [*PIN*^+^] prion aggregates can cross-seed [*PSI*^+^] formation by acting as templates on which Sup35 molecules misfold and assemble into prion aggregates (28). In the [psi^–^] [*pin*^–^] strain, Sup35 prions are not usually induced, despite the overexpression of the Sup35 or Sup35 prion domain, possibly due to the significant amyloid nucleation barrier in the absence of other prions (49). Nonetheless, research has shown that overproducing the N domain of Sup35 (Sup35-N) in [*psi*^–^ *pin*^–^] strain can occasionally elicit its assembly into liquid condensates through an LLPS-like process (54). Under hyperosmotic and energy depletion conditions, condensate formation by Sup35-N is even more pronounced upon its overproduction. On the other hand, the intracellular condensation of full-length Sup35 was usually studied with fluorophore-tagged Sup35 expressed at physiological levels in [*psi*^–^] strains using the endogenous Sup35 promoter (33, 34). In this context, Sup35 is soluble but can assemble into submicroscopic condensates under energy depletion or acidification of the cytosol. The stress-induced Sup35 condensates exhibit gel-like properties since they do not show fluorescence recovery after photobleaching, but they can be dissolved after removal of stress followed by energy restoration (33).

Our study focused on investigating how different overexpression levels of Sup35 in a yeast strain lacking preexisting prions affect the nature and the morphological characteristics of Sup35 assemblies as well as cellular growth and stress response. Our results showed that moderate overexpression of Sup35-GFP, to about fivefold to tenfold higher levels than the Sup35-GFP expressed from a centromeric plasmid under the control of the endogenous Sup35 promoter in the [*psi*^–^] YJW584 derivative strain (YJW584-ΔSup35), occasionally causes Sup35 aggregation in our yeast system (Fig. 3A; Fig. S1C and D; Table S1). Further increasing its overexpression levels can induce more extensive and significant Sup35 aggregation across cell populations. This increased aggregation, driven by higher protein concentrations, correlates with a higher fraction of insoluble Sup35 (Fig. 3H; Table S2). These intracellular insoluble aggregates do not stain positively with amyloid-specific dyes and exhibit no differences in their formation and morphology between prion-cured and non-cured strains (Fig. 3I and J; Fig. S4 and S5). Furthermore, intermediates with ribbon- or ring-like filamentous shape, typically observed during the transition into [*PSI*^+^] in [*PIN*^+^] strains, were not observed under any different overexpression levels (30). Although more assays are needed to definitively rule out [*PSI*^+^] formation, such as semi-denaturing detergent-agarose gel electrophoresis (SDD-AGE) and dependency of prion propagation on Hsp104 protein (55), it is likely that Sup35 agglomerates in our yeast system are non-amyloid, amorphous, and unstructured aggregates. Consistently, the formation of various non-amyloid assemblies by Sup35-based constructs in [*psi*^-^ *pin*^–^] cells by many studies (43, 49, 54).

It is well-established that overexpression of Sup35 or Sup35NM in [*PSI*^+^] cells can impart growth inhibition (56, 57). In [*psi*^–^ *PIN*^+^] cells, elevated levels of Sup35 also cause a growth defect (56). In both cases, Sup35 prion aggregates were formed. The lethality of Sup35 prions is likely attributed to the sequestration/inactivation of essential molecules by Sup35 prions, such as mRNA and Sup45. However, not many studies investigate whether overexpression of Sup35 in [*psi*^–^ *pin*^–^] cells, which does not lead to prion formation, could affect cellular growth. We showed that increased Sup35 overexpression in [*psi*^–^ *pin*^–^] cells can lead to a higher load of Sup35 insoluble aggregates in cells, resulting in both greater inhibition of cellular growth and an increased burden in protein production. Although no data support that non-prion Sup35 aggregates can cause a growth defect with a similar mechanism as Sup35 prion aggregates, the insoluble Sup35 aggregates in our case likely suppress cellular growth by sequestering functional Sup35 and its binding partners (e.g., Sup45 and mRNA) similarly as their prion counterparts, reducing the pool of available proteins for normal cellular functions (58). This assumption is supported by a study showing that overexpression of a disease protein TDP-43 in a yeast model can lead to the formation of non-amyloid-like TDP-43 misfolded aggregates, which are associated with cellular toxicity (42). However, we must consider the potential impact of the location of GFP within the fluorophore-tagged Sup35 construct on the protein’s behaviors and its physiological functions. It was reported that insertion of GFP between N-terminal and M domains of Sup35 (NGMC) might have a minimal interference on Sup35 aggregation and functions (59, 60). In our hand, GFP was added to the C-terminal of Sup35, which might interfere with its catalytic function as translational terminator factors and its binding with its other molecules such as translation-related molecules (e.g., Sup45 and mRNA). This might also contribute to cellular growth inhibition when they are overexpressed.

Previous data have shown fluorophore-tagged Sup35 at its physiological level can assemble into reversible condensates under stress conditions, for example, energy depletion (33), acidification of cytosol (33, 34), and hyperosmotic stress (54). Overproduction of Sup35NM or Sup35N can facilitate condensate formation under hyperosmotic stress (54). Simon Alberti’s group demonstrated these condensates can form in [*psi*^–^ *PIN*^+^] strain and conferred fitness advantage to the cells when recovering from stress compared to the [*PSI*^+^] strain, suggesting the condensate formation in the presence of Sup35 prion might be impaired and the availability of Sup35 is important for stress recovery. Our data show that under about fivefold to tenfold higher overexpression levels of Sup35-GFP driven by promoter P1 or P2, there is no significant difference in condensate formation frequency, abundance, reversibility, or physical properties compared to Sup35 at its endogenous level (Fig. 5B; Fig. S1C and D). At higher Sup35 expression levels provided by promoters P4 and P8, where significant aggregation occurs, it becomes challenging to determine if prior aggregation affects stress-induced condensation as some small aggregates resemble condensates in size and shape. Nonetheless, since stress-induced condensates generally form at a higher abundancy per cell than aggregates, we were able to identify that stress-induced condensation does occur in cells with aggregates (Fig. 5C and D). However, in cells with complete aggregate formation (cell only contains large aggregates without diffuse fluorescence and small puncta around), condensation might be suppressed due to the low availability of soluble Sup35 (Fig. S7). This suggests Sup35 aggregation might not necessarily prevent its condensation under stress conditions, possibly because, when Sup35 is saturated, its condensation is primarily dependent on the acidification of cytosol and its availability. Although Sup35 condensates have been reported to promote stress recovery, this notion was challenged by others, which showed Sup35 condensates formed under stress conditions did not confer fitness benefit (34). We show that Sup35 aggregates contribute to cellular growth inhibition and that energy depletion is more toxic to cells with higher loads of Sup35 aggregates compared to those with lower loads (Fig. 6B). However, it remains to be solved whether their different response to stress is due to Sup35 aggregation *per se* or the formation of stress-induced condensates.

In this study, we used a combinatorial approach to systematically characterize the physical properties of different states of the model protein Sup35, which exhibits complex phase behaviors in yeast cells. The method includes accessible tools for qualitative and semi-quantitative analysis, such as fluorescence microscopy, flow cytometry, super-resolution imaging, Western blot, and spot-titer growth assays. We found notable differences between stress-induced Sup35 condensates and large non-prion aggregates. FM and SRRF images revealed that Sup35 condensates are smaller, rounder, smoother at the periphery, and more abundant per cell than large aggregates (Fig. 7), consistent with previous studies. Furthermore, Sup35 condensates within and between cells showed similar physical properties (size, shape, and abundance), while aggregates displayed significant cell-to-cell variations. A key distinction between condensates and aggregates is their reversibility: stress-induced condensates dissolve after stress removal, whereas aggregates persist and can impair growth recovery. However, flow cytometry and pixel intensity analysis did not reveal clear differences in fluorescence distribution, highlighting their limitations in distinguishing similar assemblies.

The integrated methodology used in the study can uncover differences in physical properties and reversibility between assembled states of Sup35, showcasing the potential of employing this approach to systematically characterize other IDR-containing proteins with unexplored phase behaviors. However, the method has several challenges and limitations. First, there is no unifying rule that clearly defines which specific morphological features characterize these assemblies. We also acknowledge that, without prior knowledge about Sup35, it becomes unfeasible to differentiate between Sup35 assemblies based on their appearance or structure. Their distinction becomes clear only when Sup35 condensates are found to be reversible and condensate formation can improve cellular fitness from stress, while Sup35 aggregates are irreversible and lead to defects in both cellular growth and stress recovery. Second, many findings in the study are based on observation with microscopy, which may have inherent limitations in resolution. This can impact the capability to distinguish the details between different assemblies. Such image artifacts can complicate the interpretation of the observation. Therefore, examination of physical properties *in vivo* alone cannot fully define the nature of cellular assemblies; rather, one should pair *in vivo* analysis with biochemical assays and an examination of the biological function of assemblies and their reversibility to discern the nature of biomolecular structures. That Sup35 shows a metastability in its condensation behavior is important to consider when assessing its biological functions by probing its expression level artificially. In itself, such metastability can be explained based on general observations of protein phase behavior.

## MATERIALS AND METHODS

### Plasmid construction

DNA sequences encoding Sup35-GFP and Venus were obtained from Genscript and cloned into in-house plasmids provided by Dr. Dominik Mojzita (VTT Technical Research Centre of Finland, Espoo, Finland) using *Pac*I and *Xho*I restriction enzymes in their multiple cloning sites (36). The expression cassette of the genes of interest would be released using the *Not*I restriction enzyme prior to the transformation and then integrated into the yeast genome into the *LEU2* locus. The expression cassette contains homologous recombination sites for the integration in *LEU2* locus, a synthetic promoter, an open reading frame of a gene, and a terminator. The synthetic promoters consist of an *ENO1* core promoter (*ENO1*cp) and different number of binding sites specific for a synthetic transcriptional factor (sTF), PhIF. The binding sites were engineered to be upstream of the (*ENO1*cp). The promoters were engineered to contain zero, one, two, four, or eight binding sites, providing a board range of protein expression levels in *S. cerevisiae*. All plasmids were transformed into chemically competent *E. coli* Top10 cells for amplification using the standard heat shock method. A positive colony from the LB agar plates supplemented with 100 µg/mL ampicillin was selected for plasmid purification. All constructed plasmids were verified by Sanger sequencing (Eurofins Genomics). Information of plasmids with different promoter strengths and gene of interest is listed in the Tables S7 through S9.

### Plasmid transformation and protein expression in yeast

Plasmids were transformed into a yeast strain CEN.PK102-5B (*ura3-52*, *his3-delta1*, *leu2-3,-112*, *TRP1*, *MAL2-8c*, *SUC2*), which was provided by Dr. Dominik Mojzita, using a standard lithium acetate method (61). The expression cassette of a transcription factor, PhIF, was already transformed into the yeast genome into the *URA3* locus. The *TDH3* core promoter was used to ensure low and constitutive expression of PhIF, which can bind to its cognate binding site already engineered on the promoters of the expression cassette of a target gene. Yeast transformation mixtures were plated on synthetic dropout (SD) medium lacking leucine and uracil (SD-Leu-Ura). The medium was made with 6.7 g/L yeast nitrogen base without amino acid (Sigma Aldrich), 20 g/L D-glucose (Sigma Aldrich), 10 g/L agarose (Sigma Aldrich) if needed, and 690 mg/L complete supplement mixture without leucine and uracil (CSM-Leu-Ura, Formedium) for the selection of auxotrophic transformants. A positive colony was picked and grown at 30°C in SD-Leu-Ura medium.

### Energy depletion and restoration of yeast

To arrest growth and induce energy depletion of yeast cells, exponentially growing *S. cerevisiae* cells were harvested and incubated in the SD-Leu-Ura medium for 3–4 hours, in which D-glucose was replaced by 20 mM 2-deoxyglucose (Sigma Aldrich) for the suppression of glycolysis and 10 mM antimycin A (Sigma Aldrich) was supplemented for the inhibition of mitochondrial ATP production (33, 62). The cells treated with the combination of these two chemicals effectively diminish up to 95% of cellular ATP production (63). To restore the growth and metabolism of stress-exposed cells, the concentrated cells were washed three times with sterilized 1 x phosphate-buffered saline solution (pH 7.4) (Gibco) and then incubated in SD-Leu-Ura medium with 2% glucose.

### Protein extraction from yeast and immunoblotting

*S. cerevisiae* was grown at 30°C with a starting OD_600_ = 0.2 in SD-Leu-Ura medium for 20 hours with 220 rpm shaking. An equivalent of 10 OD_600_ amount of cells was collected through centrifugation and rapidly frozen. Cells were resuspended in lysis buffer (20 mM Tris-HCl, 1 M KCl, 2 mM EDTA, 1 mM DTT, and pH 7.5) supplemented with cOmplete Protease Inhibitor Cocktail (Roche). Cells with the addition of 0.5-mm glass beads (Thermo Fisher Scientific) were lysed with Disruptor genie (Scientific Industries, USA) in three 1-min pulses with 5-min incubation on ice in between. Cell lysates were cleared of unbroken cells and glass beads by low-speed centrifugation (500 x *g*) for 2 minutes. Then, the cell lysate mixture was collected and labeled as the whole-cell extract. The supernatant of the whole-cell extract was collected after centrifugation at full speed for 20 minutes. All centrifugation steps were carried out at 4°C. Equal volumes of both the whole-cell extract and the supernatant of each sample were run on precast SDS-polyacrylamide gels (4%–15% Mini-PROTEAN TGX Precast Protein Gels, BIORAD). Proteins from the gels were transferred onto a nitrocellulose membrane (Amersham, pore size 0.45 µm) by electroblotting (25 V, 30 minutes), followed by pre-blocking with 5% nonfat milk and probing with antibody and detection with Pierce ECL Western Blotting Chemiluminescent Substrate (Thermo Fisher Scientific). The antibody used for detecting Venus and Sup35-GFP is GFP polyclonal antibody conjugated with horseradish peroxidase (Thermo Fisher Scientific). For detecting yeast glucose-6-phosphate dehydrogenase (G6PDH) (Thermo Fisher Scientific), G6PDH polyclonal antibody conjugated with HRP was used.

To compare protein levels of each sample, protein bands from immunoblotting membranes were quantified with densitometry using ImageJ Fiji (version 1.47d). G6PDH was selected as a loading control, and its intensity was used to normalize the intensity of each protein band. Data were expressed as the ratio of the target protein to the loading control, and results were averaged from three independent experiments. Final values were normalized to the amount of Sup35-GFP without overexpression, setting this baseline to 1. Sup35-GFP without overexpression was expressed from a YJW584 derivative strain with a deletion of the chromosomal *Sup35* gene, YJW584-ΔSup35 (33). In this strain, Sup35-GFP was expressed from a centromeric plasmid under the control of the endogenous Sup35 promoter ( Table S8). The YJW584 strain (*MAT*a, *leu2-3*,−*112; his3-11*,*15; trp1-1; ura3-1; ade1-14; can1-100*, [*PIN*^+^], [*psi*^–^]) was originally from Jonathan S. Weissman’s lab (44).

### Fluorescence microscopy and image analysis

Cell cultures from different cultivation conditions were placed between a polylysine-coated glass slide and a coverslip and imaged on a Zeiss Axio Observer Z1 microscope with a 100 x/1.4 oil immersion objective and an Andor iXon Ultra 888 camera. The GFP fluorescence was observed using excitation light at 470  nm (Zeiss Colibri, 25% intensity), and the emitted light was collected at 515–535 nm with an exposure time of 100 ms. Phase contrast and Z-stack ﬂuorescence images were acquired by Zeiss ZEN Pro software and further analyzed with ImageJ Fiji software (version 1.47d). For pixel intensity analysis, the histogram of the intensity of fluorescence channel was extracted from microscopy images (8-bit) using ImageJ Fiji. The counts of each intensity value were normalized by the number of cells. The intensity values and the normalized counts of each intensity value were converted to logarithmic values for better visualization purpose. A log_2_–log_10_ graph was created with Origin software to describe the pixel distribution of samples.

### Super-resolution radial fluctuations (SRRF)

To collect data for SRRF analysis, samples were prepared as for fluorescence microscopy and imaged similarly as above, with the exception that a sequential series of 100 images were acquired and the exposure time was varied depending on the fluorescence intensity of the samples. SRRF analysis of a given data set was performed using the ImageJ plugin (NanoJ-SRRF) with the following setting: ring radius of 1 pixel for radiality calculations, radiality magnification of 5, axes in ring of 6, and Temporal Radiality Average (TRA) for the temporal analysis of radiality images (52).

### Microfluidic chip production and time-lapse imaging

The dynamics and the localization of intracellular assemblies produced by different proteins over time were analyzed by monitoring individual cells placed on microfluidic chips. The preparation of microfluidic chips was done using the same protocol as previously reported (64). In the microfluidic chips, yeast cells were growing in 100 × 100 µm pockets with a height of 5 µm. Fresh media were constantly supplying for the cells.

Cells were grown in microfluidic chips for 2–3 hours before switching to energy-depletion media. The microfluidic chips were placed in a chamber that can keep the temperature at 30°C, and the media ﬂow rate was kept at 300 µm/s in both main channels using an automated syringe pump. A series of phase contrast and fluorescence images of an interesting region was taken in a 15- or 20 minute interval for 2–3 hours with the same microscopic instrument described as above.

### Flow cytometry

Yeast cells were cultured for 20 hours at 30°C with a shaking speed of 220 rpm. Cells were then harvested by centrifugation and resuspended in 1 x PBS buffer pH 7.4. Flow cytometry measurements were performed on a BD FACSAria II cytometer (BD Biosciences) using 488-nm laser for excitation and a 530-/30-nm bandpass filter for emission detection. A total of 10000 events per sample were recorded. Data analysis was performed using the Flowjo software (https://www.flowjo.com/).

### Spot-titer growth assay

*S. cerevisiae* cells were grown for 20 hours at 30°C with shaking (220 rpm). Cell cultures were first diluted to OD_600_ = 0.1, after which cell cultures were further diluted with fourfold serial dilutions. Samples with different dilutions were spotted on SD-Leu-Ura agar plates. Plates were placed in the 30°C incubator for 24 hours and then photographed with a camera from the Molecular Imager Gel Doc XR+ (BioRad) operating at the colorimetric mode against the black background. To study how stressed cells recover from stress, the energy-depleted cells were harvested by centrifugation (3900 rpm, 2 minutes) and then washed three times with 1 x PBS buffer (pH 7.4). Later, cells were resuspended with fresh SD-Leu-Ura liquid medium and grown for 2 hours at 30°C with shaking. Cell cultures were diluted with fourfold serial dilutions and spotted on the SD-Leu-Ura agar plates. Plates were placed in a 30°C incubator and were photographed 24 hours after spotting.

### Induction of [*PSI*^+^] *de novo*

A galactose-inducible plasmid encoding Sup35-GFP was transformed into the YJW584 strain (*MAT*a, *leu2-3*,−*112; his3-11*,−*15; trp1-1; ura3-1; ade1-14; can1-100*, [*PIN*^+^], [*psi*^–^]), a gift from Prof. Juha Saarikangas (University of Helsinki). The YJW584 strain was originally from Jonathan S. Weissman’s lab (44). Yeast cells with the Sup35 overexpression plasmids were grown in Sraf-Leu medium containing 2% raffinose for 20 hours at 30°C with shaking (220 rpm). The Sgal-Leu medium containing 2% galactose was used to replace Sraf-Leu and added to culture with the OD_600_ of 0.5 to induce *de novo* Sup35 aggregation for 2 days. To increase the likelihood and the frequence of prion formation, fresh Sgal-Leu medium was added to cell cultures for growing for 3 additional days. Cells were spotted on a YPD plate to determine the prion formation.

### Staining of yeast cells with amyloid-specific dyes

Yeast cells were stained with thioflavin T using a previously published protocol (42). The yeast cultures overexpressing Sup35 with SynPro were grown in SD-Leu-Ura medium for 20 hours at 30°C with shaking (220 rpm). Cells were harvested to achieve a final OD 600 of 0.25–1.0 and fixed in 5 mL of 50 mM KPO₄ (pH 6.5), 1 mM MgCl₂, and 4% formaldehyde for 2 hours. After fixation, the cells were washed with 5 mL of PM [0.1 M KPO₄ (pH 7.5), 1 mM MgCl₂] and resuspended in PMST [0.1 M KPO₄ (pH 7.5), 1 mM MgCl₂, 1 M sorbitol, 0.1% Tween 20] to a final OD 600 of 10. One hundred microliters of the cell suspension was incubated with 0.6 µL of 2-mercaptoethanol and 1 mg/mL zymolyase for 15 minutes. The resulting spheroplasted cells were washed once in PMST and resuspended in 100 µL of PMST. The cells were then incubated with 0.005% thioflavin T (Thermo Fisher Scientific) and 0.1% amytracker 630 (Ebba Biotech AB) for 20 minutes at room temperature, washed three times with PMST, and examined using the Zeiss Axio microscope. Sup35-GFP was visualized in the GFP channel, while thioflavin T staining was observed in the CFP channel and amytracker 630 was in the mCherry channel. To minimize the crosstalk between GFP and CFP, the following settings were used. The GFP signal was acquired using excitation light at 470 nm, while collecting the emitted light of 515–535 nm. The CFP signal was acquired using excitation light at 420 nm, while collecting the emitted light of 461–485 nm. The mCherry signal was acquired using excitation light at 590 nm, while collecting the emitted light of 615–675 nm.

### Prion curing of yeast strain

The yeast strain CEN.PK102-5B of SynPro was cured of prions by streaking onto a YPD plate containing 5 mM guanidine HCl (GuHCl) where they were grown for 3 days. GuHCl is a potent chaotropic agent known for its ability to eradicate prions by disabling HSP104 (65). To increase the probability of prion elimination, strains were subjected to three more passes on GuHCl media and were then transferred to YPD media for colony purification. All plasmids were transformed into the cured strain using the transformation protocol as described above.

## SUPPLEMENTAL MATERIAL

Supplemental material aem.01703-24-s0001.pdf Figures S1 to S9; Tables S1 to S9.

ASM does not own the copyrights to Supplemental Material that may be linked to, or accessed through, an article. The authors have granted ASM a non-exclusive, world-wide license to publish the Supplemental Material files. Please contact the corresponding author directly for reuse.
