# Supplementary material for: Effects of Sup35 overexpression on the formation, morphology, and physiological functions of intracellular Sup35 assemblies
Source: Appl Environ Microbiol. 2025 Feb 6;91(3):e01703-24. doi: 10.1128/aem.01703-24 (PMC11921396; doi:10.1128/aem.01703-24)
Supplement: Supplemental material — Figures S1 to S9; Tables S1 to S9. [file aem.01703-24-s0001.pdf]

Supplementary data:

Effects of Sup35 overexpression on the formation, morphology, and physiological functions of intracellular Sup35 assemblies

Jianhui Feng[1,3], Ekaterina Osmekhina[1,3], Jaakko V.I. Timonen[2,3], Markus B. Linder[1,3]

[1]Department of Bioproducts and Biosystems, School of Chemical Engineering, Aalto University, Espoo 02150, Finland

[2]Department of Applied Physics, School of Science, Aalto University, Espoo 02150, Finland

[3]The Center of Excellence in Life-Inspired Hybrid Materials (LIBER), Aalto University, Espoo 02150, Finland

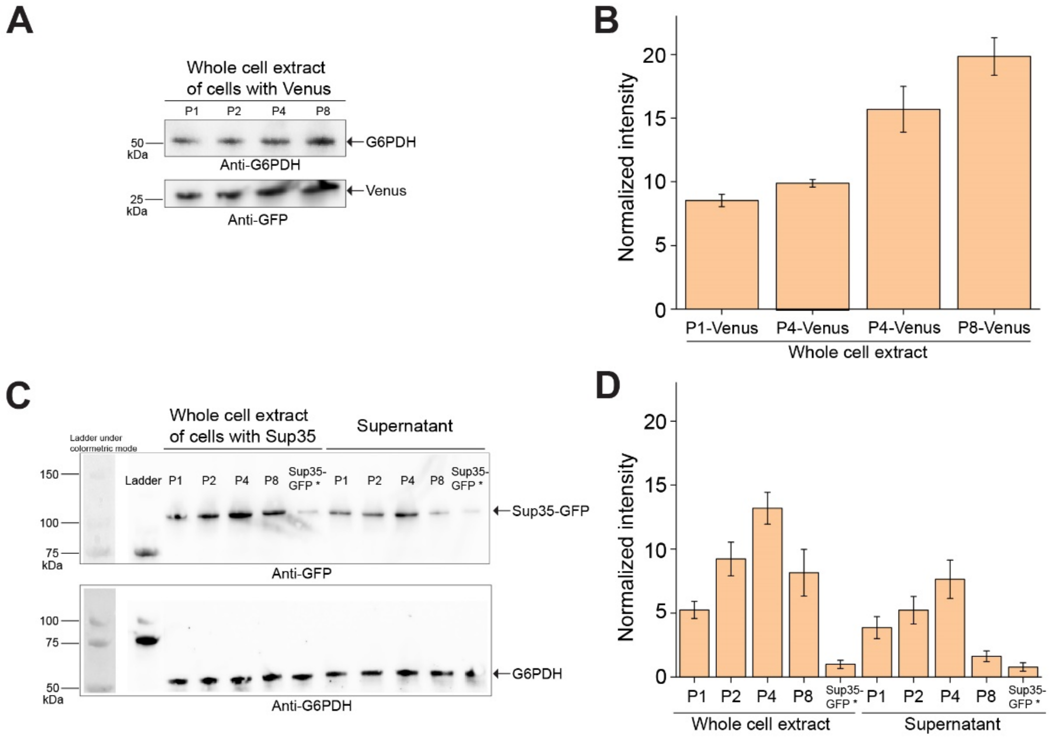

Figure S1. Comparison of protein levels for Sup35-GFP and Venus. (**A**) Respresentative western blot results for Venus expressed by the SynPro system under different promoter strengths. After removal of unbroken cells and glass beads by mild centrifugation, the supernatant of cell lysates (labed as whole cell extracts) were used for blotting Venus which was detected by anti-GFP antibody, with G6PDH used as a loading control, which was detected by anti-G6PDH antibody. (**B**) Comparison of normalized levels for Venus shown on the panel (A) using densitometry. All cell cultures with different promoter strengths were grown under identical condition. (**C**) Respresentative western blot results for Sup35-GFP expressed by the SynPro system under different promoter strengths. The Sup35-GFP* indicates Sup35-GFP expressed from a [*psi*-] YJW584 derivative strain (YJW584-ΔSup35), where the chromosomal Sup35 gene was deleted and the expression of Sup35-GFP is controlled from a centromeric plasmid under the control of the endogenous Sup35 promoter. Whole cell extracts and supernatant after cell lysis were collected and used for blotting to compare the soluble fraction of Sup35 from different cell cultrues. Anti-GFP antibody was used for detecting Sup35-GFP, with G6PDH used as a loading control. (**D**) Comparison of normalized levels for Sup35-GFP shown on the panel (C) using densitometry. All data from panels (A) and (D), were normalized to the amount

of Sup35-GFP* without overproduction, with this amount set to 1. Data used for ploting panels (B) and (D) can be found in Table S1.

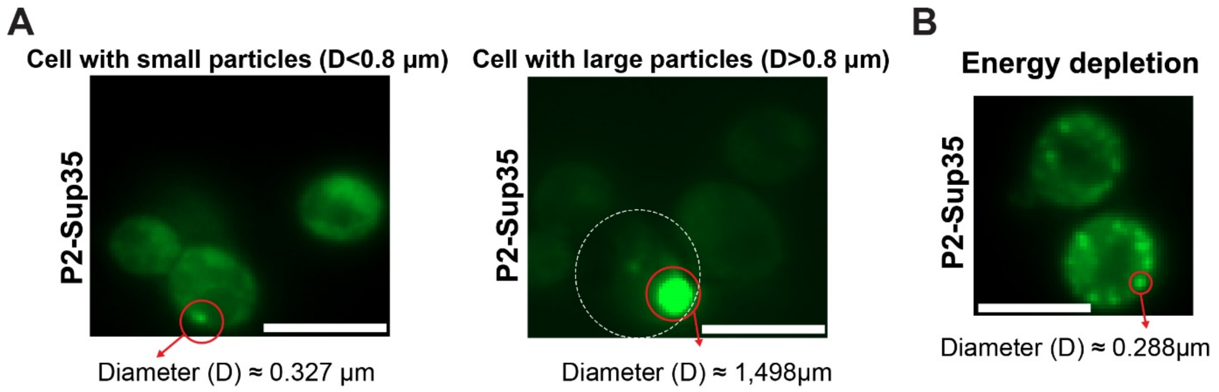

Figure S2. Size of intracellular Sup35 assemblies. (**A**) Representative images of P2-Sup35 cells bearing different sizes of particles. White dashed circle indicates cell border. (**B**) Representative image of P2-Sup35 cells bearing stressed induced condensates. *Scale bars: 5 μm.*

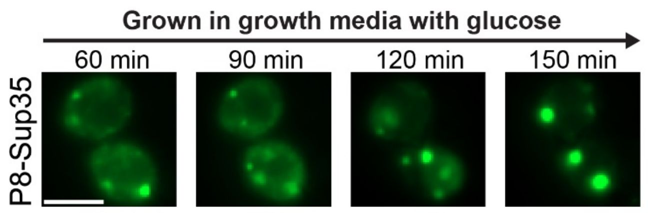

Figure S3. FM images taken from a time-lapse experiment of *S. cerevisiae* expressing P8-Sup35. Exponentially growing cells were transferred to a microfluidic device and grown in fresh media containing glucose for 150 minutes. Scale bar: 5 μm.

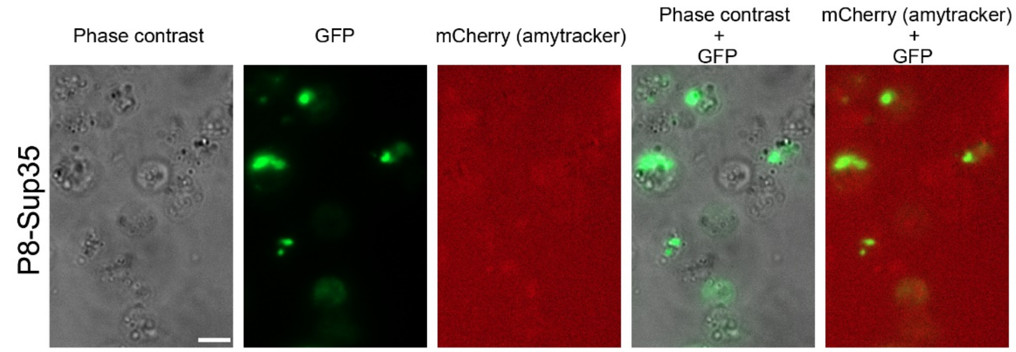

Figure S4. Yeast cells expressing P8-Sup35 were stained with amytracker to assess amyloid formation. P8-Sup35 did not contain amyloid-like structures as there was no fluorescence signal colocalization between Sup35 aggregates and amytracker (amytracker fluorescence signal was visualized in mCherry channel). Scale bar: 5 µm.

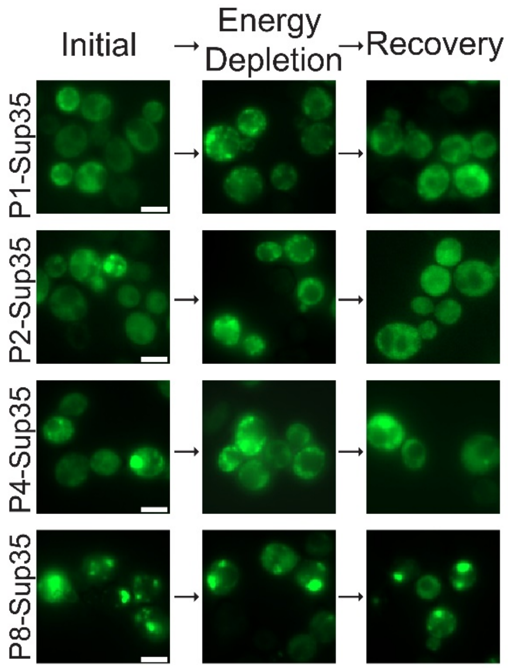

Figure S5. *S. cerevisiae* cells were cured of prions expressing Sup35-GFP under different promoter strengths during exponential growth and during energy depletion and after resupplying cells with fresh media. Scale bars of all FM images are 5 µm.

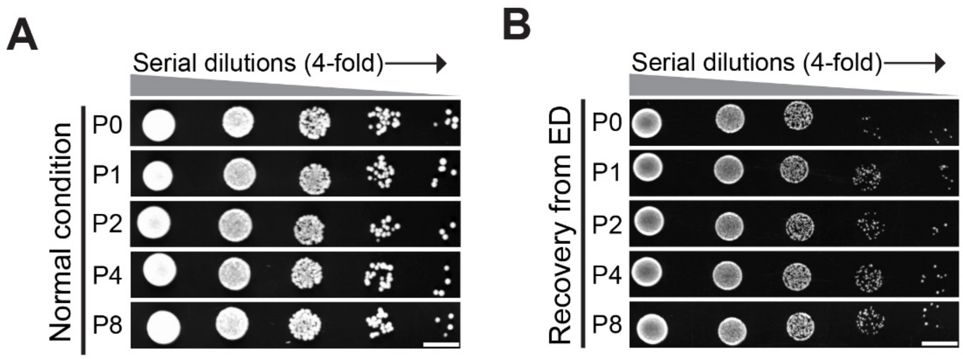

Figure S6. Spot-titer growth assay. (**A**) Spot-titer growth assay of cells grown in fresh media with different overexpression levels of Venus. (**B**) Spot-titer growth assay of cells with different overexpression levels of Venus that were recovered by resupplying cells with fresh media after energy depletion. All cells were grown under the identical condition with the same *starting* OD_{600}. Scale bars: 1 cm.

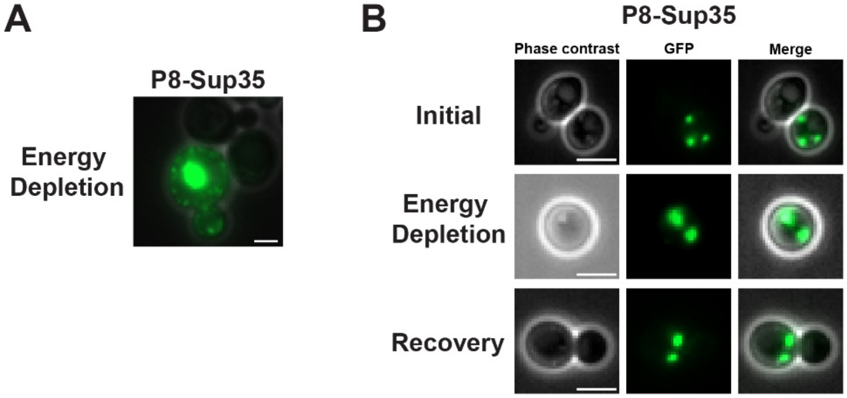

Figure S7. Fluorescence micrographs of *S. cerevisiae* expressing P8-Sup35 under different conditions. (**A**) Representative images of a P8-Sup35 cell containing large aggregates and multiple small particles under energy depletion condition. (**B**) Representative images of P8-Sup35 cells containing only large aggregates. These cells can be observed under initial condition prior to stress, energy depletion, and stress recovery condition. Scale bars of all FM images are 5 µm.

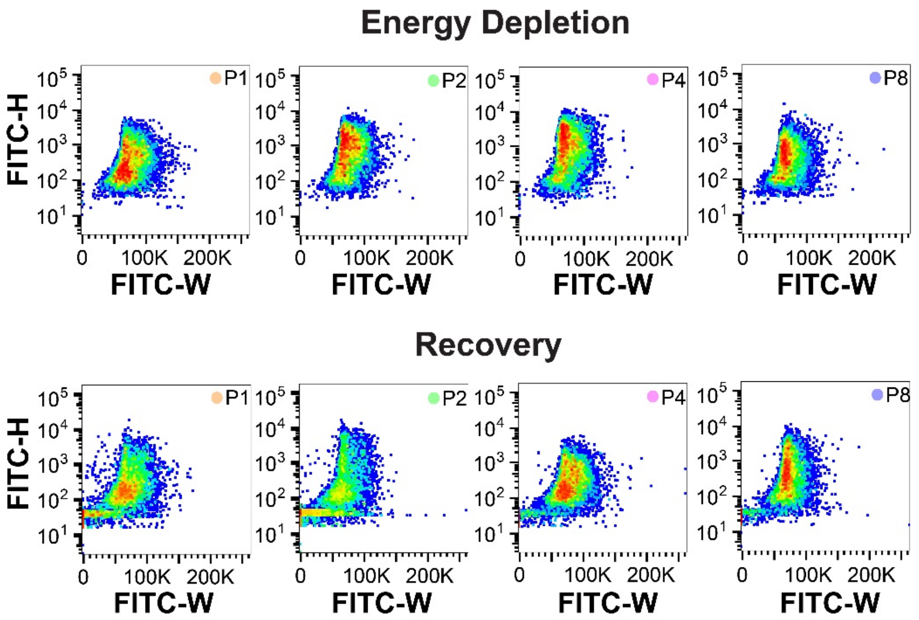

Figure S8. FITC-H versus FITC-W scatter plots of cell cultures with different expression levels of Sup35-GFP under different conditions. All scatter plots were derived from 10000 events.

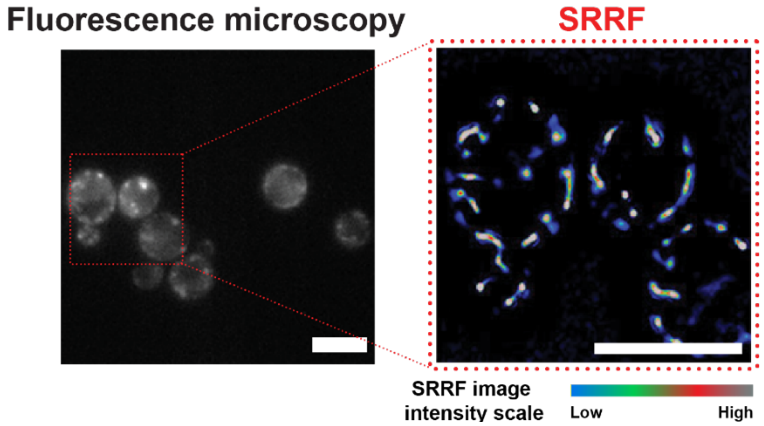

Figure S9. The magnified version fluorescence microscopy image of *S. cerevisiae* and the corresponding image processed by super-resolution radial fluctuations (SRRF) analysis. SRRF image circled by a red dashed box represents the region (marked with a red dashed box) of the fluorescence microscopy image. The fluorescence intensity of the fluorophore in the SRRF image was pseudo-color coded, with grey representing the highest intensity, followed by red, green, and blue representing intensity in decreasing order. The yeast cells expressing P1-Sup35 under energy depletion was shown as an example. All scale bars are 5 μm.

Table S1. Comparison of protein levels expressed by different promoter strengths using densitometry. (Data for Fig. S1)

Protein	Fraction	Number of repeats	Mean	SD
P1-Sup35-GFP	Whole cell extract	3	5.25	0.67
P2-Sup35-GFP	Whole cell extract	3	9.23	1.31
P4-Sup35-GFP	Whole cell extract	3	13.69	1.24
P8-Sup35-GFP	Whole cell extract	3	8.15	1.82
Sup35-GFP*	Whole cell extract	3	1.00	0.32
P1-Sup35-GFP	Supernatant	3	3.87	0.86
P2-Sup35-GFP	Supernatant	3	5.22	1.07
P4-Sup35-GFP	Supernatant	3	6.93	1.49
P8-Sup35-GFP	Supernatant	3	1.63	0.42
Sup35-GFP*	Supernatant	3	0.79	0.33
P1-Venus	Whole cell extract	3	8.37	0.49
P2-Venus	Whole cell extract	3	9.99	0.29
P4-Venus	Whole cell extract	3	16.37	1.80
P8-Venus	Whole cell extract	3	19.84	1.49

Sup35-GFP*: The protein was expressed from a derivative of [*psi*$^{-}$ *PIN*$^{+}$] YJW584 strain (YJW584-ΔSup35), where the chromosomal Sup35 gene was deleted and Sup35-GFP was expressed from a centromeric plasmid under the control of the Sup35 endogenous promoter.

Table S2. Comparison of soluble fraction of Sup35-GFP expressed by different promoter strengths. Data was calculated based on the Table S3. (Data for Fig. 3H.)

Protein	Number of repeats	Mean of soluble fraction (%)	SD
P1-Sup35-GFP	3	73.81	13.01
P2-Sup35-GFP	3	56.27	6.39
P4-Sup35-GFP	3	57.49	8.04
P8-Sup35-GFP	3	19.99	3.59

Table S3. Effect of different overexpression levels on the formation of fluorescent particles by Sup35-GFP with the SynPro system. (Data for Fig. 3B-3D.)

**Proteins**		**Cells with particles**		**Cells with particles (D>0.8 µm)**		**Cells with >4 particles**		**Total**
	Culture	Number	%	Number	%	Number	%	
1-Sup35-GFP	1	12	2.33	0	0.00	0	0.00	516
	2	16	3.94	0	0.00	0	0.00	406
	3	4	0.69	0	0.00	0	0.00	580
	4	8	1.67	0	0.00	0	0.00	480
	5	12	1.96	0	0.00	0	0.00	612
	6	8	1.50	2	0.38	0	0.00	532
	**Average**		**2.01**		**0.06**		**0.00**	
	**SD**		**1.00**		**0.14**		**0.00**	
2-Sup35-GFP	1	16	4.23	4	1.06	0	0.00	378
	2	12	3.35	6	1.68	2	0.56	358
	3	16	4.79	2	0.60	2	0.60	334
	4	16	5.19	2	0.65	4	1.30	308
	5	20	7.04	0	0.00	0	0.00	284
	6	20	4.33	2	0.43	0	0.00	462
	7	12	4.92	2	0.82	2	0.82	244
	**Average**		**4.84**		**0.75**		**0.47**	
	**SD**		**1.06**		**0.49**		**0.46**	
4-Sup35-GFP	1	56	14.81	44	11.64	24	6.35	378
	2	108	37.11	93	31.96	66	22.68	291
	3	88	32.35	72	26.47	42	15.44	272
	4	124	28.05	88	19.91	60	13.57	442
	5	128	25.00	66	12.89	40	7.81	512
	6	70	16.13	48	11.06	30	6.91	434
	**Average**		**25.58**		**18.99**		**12.13**	
	**SD**		**8.07**		**7.95**		**5.83**	
8-Sup35-GFP	1	210	48.84	192	44.65	50	11.63	430
	2	184	34.07	176	32.59	56	10.37	540
	3	184	40.89	92	20.44	30	6.67	450
	4	232	49.15	184	38.98	72	15.25	472
	5	88	34.92	72	28.57	42	16.67	252
	6	156	41.27	118	31.22	52	13.76	378
	**Average**		**41.52**		**32.74**		**12.39**	
	**SD**		**5.93**		**7.66**		**3.31**	

Table S4. Comparison of percentage of cells with specific fluorescent pattern in cells overproducing Sup35 at different levels. (Data for Fig. 3G.)

Protein		Cells without particles				Cells with particles						
	Culture	I		II		III		IV		V		Total
		Num-ber	%	Num-ber	%	Num-ber	%	Num-ber	%	Num-ber	%	
P1-Sup35-GFP	1	6	1.16	498	96.51	12	2.33	0	0.00	0	0.00	516
	2	8	1.97	382	94.09	16	3.94	0	0.00	0	0.00	406
	3	16	2.76	560	96.55	4	0.69	0	0.00	0	0.00	580
	4	10	2.08	462	96.25	8	1.67	0	0.00	0	0.00	480
	5	12	1.96	600	98.04	10	1.63	2	0.33	0	0.00	612
	6	12	2.26	520	97.74	8	1.50	0	0.00	0	0.00	532
	**Average**		**2.03**		**96.53**		**1.96**		**0.05**		**0.00**	
	**SD**		**0.47**		**1.28**		**1.01**		**0.12**		**0.00**	
P2-Sup35-GFP	1	16	4.23	346	91.53	12	3.17	4	1.06	0	0.00	378
	2	14	3.91	344	96.09	6	1.68	4	1.12	2	0.56	358
	3	10	2.99	308	92.22	12	3.59	2	0.60	2	0.60	334
	4	18	5.84	274	88.96	14	4.55	2	0.65	0	0.00	308
	5	24	8.45	240	84.51	20	7.04	0	0.00	0	0.00	284
	6	20	4.33	422	91.34	18	3.90	2	0.43	0	0.00	462
	7	4	1.64	228	93.44	10	4.10	2	0.82	0	0.00	244
	**Average**		**4.49**		**91.16**		**4.00**		**0.67**		**0.17**	
	**SD**		**2.01**		**3.38**		**1.50**		**0.36**		**0.26**	
P4-Sup35-GFP	1	34	8.99	290	76.72	12	3.17	34	8.99	10	2.65	378
	2	12	4.12	171	58.76	36	12.37	48	16.49	24	8.25	291
	3	16	5.88	148	54.41	22	8.09	46	16.91	20	7.35	272
	4	30	6.79	288	65.16	18	4.07	84	19.00	32	7.24	442
	5	46	8.98	338	66.02	40	7.81	64	12.50	24	4.69	512
	6	34	7.83	390	89.86	16	3.69	40	9.22	14	3.23	434
	**Average**		**7.10**		**68.49**		**6.53**		**13.85**		**5.57**	
	**SD**		**1.74**		**11.79**		**3.26**		**3.87**		**2.16**	
P8-Sup35-GFP	1	44	10.23	216	50.23	76	17.67	68	15.81	66	15.35	430
	2	50	9.26	306	56.67	52	9.63	78	14.44	54	10.00	540
	3	30	6.67	236	52.44	34	7.56	82	18.22	68	15.11	450
	4	64	13.56	176	37.29	40	8.47	138	29.24	54	11.44	472
	5	38	15.08	126	50.00	16	6.35	56	22.22	16	6.35	252
	6	34	8.99	188	49.74	30	7.94	86	22.75	40	10.58	378
	**Average**		**10.63**		**49.39**		**9.60**		**20.45**		**11.47**	
	**SD**		**2.85**		**5.92**		**3.74**		**4.97**		**3.10**	

Table S5. Effect of condition on the formation of fluorescent particles by Sup35-GFP expressed at different levels with the SynPro system. (Data for Fig. 5B.)

Protein	Initial				Energy depletion				Recovery			
	Culture	With particles		Total	Culture	With particles		Total	Culture	With particles		Total
		Number	%			Number	%			Number	%	
P1-Sup35-GFP	1	12	2.33	516	1	222	52.86	420	1	36	8.87	406
	2	16	3.94	406	2	368	79.65	462	2	30	5.86	512
	3	4	0.69	580	3	210	54.69	384	3	34	6.69	508
	4	8	1.67	480	4	182	68.94	264	4	20	4.85	412
	5	12	1.96	612	5	350	65.54	534	5	36	9.18	392
	6	8	1.50	532	6	264	65.02	406	6	48	8.99	534
	**Average**		**2.01**				**64.45**				**7.41**	
	**SD**		**1.00**				**8.97**				**1.69**	
P2-Sup35-GFP	1	16	4.23	378	1	410	81.03	506	1	90	20.83	432
	2	12	3.35	358	2	262	62.09	422	2	44	9.44	466
	3	16	4.79	334	3	336	78.50	428	3	34	7.87	432
	4	16	5.19	308	4	324	78.64	412	4	32	8.12	394
	5	20	7.04	284	5	284	75.94	374	5	64	12.50	512
	6	20	4.33	462	6	344	81.90	420	6	34	6.97	488
	7	12	4.92	244	7	290	76.72	378	7	42	9.46	444
	**Average**		**4.84**				**76.4**				**10.74**	
	**SD**		**1.06**				**6.17**				**4.43**	
P4-Sup35-GFP	1	56	14.81	378	1	362	88.29	410	1	204	48.34	422
	2	108	37.11	291	2	354	75.64	468	2	88	21.57	408
	3	88	32.35	272	3	312	77.23	404	3	112	25.57	438
	4	124	28.05	442	4	272	71.20	382	4	154	32.91	468
	5	128	25.00	512	5	328	78.10	420	5	170	37.28	456
	6	70	16.13	434	6	356	80.91	440	6	246	44.57	552
	**Average**		**25.58**				**78.56**				**35.04**	
	**SD**		**8.07**				**5.24**				**9.56**	
P8-Sup35-GFP	1	210	48.84	430	1	272	67.33	404	1	248	59.33	418
	2	184	34.07	540	2	130	38.92	334	2	166	40.69	408
	3	184	40.89	450	3	242	54.02	448	3	228	44.53	512
	4	232	49.15	472	4	254	60.77	418	4	208	52.53	396
	5	88	34.92	252	5	224	56.57	396	5	266	55.42	480
	6	156	41.27	378	6	138	40.12	344	6	312	58.43	534
	**Average**		**41.52**				**52.95**				**51.82**	
	**SD**		**5.93**				**10.36**				**6.96**	

Table S6. Comparison of percentage of cells with specific fluorescent pattern in cells overproducing Sup35 at different levels. (Data for Fig. 5D.)

Protein		Initial			Energy depletion			Recovery		
		Cells with large aggregates and >8 small particles		Total cells with large aggregates	Cells with large aggregates and >8 small particles		Total cells with large aggregates	Cells with large aggregates and >8 small particles		Total cells with large aggregates
	Culture	Number	%		Number	%		Number	%	
P4-Sup35-GFP	1	11	11,11 %	99	132	57.14	231	11	12,50 %	88
	2	12	13,64 %	88	33	60.00	55	22	18,18 %	121
	3	11	4,55 %	242	110	62.50	176	22	13,33 %	165
	4	10	3,60 %	278	99	52.94	187	33	13,64 %	242
	5	22	7,14 %	308	121	47.83	253	11	10,00 %	110
	**Average**		**8.01**			**56.08**			**13.53**	
	**SD**		**3.83**			**5.21**			**2.65**	
P8-Sup35-GFP	1	8	5.56	144	54	60.00	90	45	26.32	171
	2	8	8.33	96	81	56.25	144	27	18.75	144
	3	12	10.34	116	108	54.55	198	18	20.00	90
	4	8	7.14	112	72	61.54	117	45	27.78	162
	5	4	5.26	76	54	50.00	108	36	20.00	180
	**Average**		**7.33**			**56.47**			**22.5**	
	**SD**		**1.87**			**4.09**			**3.71**	

Table S7. Amino acid sequences of proteins used in this study.

Protein	Amino acid sequence
Venus	MWSHPQFEKSKGEELFTGVVPILVELDGDVNGHKFSVSGEGEGDATYGKLTLKLICTTGKLPVPWPTLVTTL<br>GYGLQCFARYPDHMKQHDFFKSAMPEGYVQERTIFFKDDGNYKTRAEVKFEGDTLVNRIELKGIDFKEDG<br>NILGHKLEYNYNSHNVYITADKQKNGIKANFKIRHNIEDGGVQLADHYQQNTPIGDGPVLLPDNHYLSYQS<br>ALSKDPNEKRDHMVLLEFVTAAGITHGMDELYKGS
Sup35-GFP*	MSDSNQGNNQQNYQQYSQNGNQQQGNNRYQGYQAYNAQAQPAGGYYQNYQGYSGYQQGGYQQY<br>NPDAGYQQQYNPQGGYQQYNPQGGYQQQFNPQGGRGNYKNFNYNNNLQGYQAGFQPQSQGMSLN<br>DFQKQQKQAAPKPKKTLKLVSSSGIKLANATKKVGTKPAESDKKEEEKSAETKEPTKEPTKVEEPVKKEEKPV<br>QTEEKTEEKSELPKVEDLKISESTHNTNNANVTSADALIKEQEEEVDDEVVNDMFGGKDHVSLIFMGHVD<br>AGKSTMGGNLLYLTGSVDKRTIEKYEREAKDAGRQGWYLSWVMDTNKEERNDGKTIEVGKAYFETEKRR<br>YTILDAPGHKMYVSEMIGGASQADVGVLVISARKGEYETGFERGGQTREHALLAKTQGVNKMVVVVNK<br>MDDPTVNWSKERYDQCVSNVSNFLRAIGYNIKTDVVFMPVSGYSGANLKDHVDPKECPWYTGPTLLEYL<br>DTMNHVDRHINAPFMLPIAAKMKDLGTIVEGKIESGHIKKGQSTLLMPNKTAVEIQNIYNETENEVDMA<br>MCGEQVKLRIKGVEEEDISPGFVLTSPKNPIKSVTKFVAQIAIVELKSIIAAGFSCVMHVHTAIEEVHIVKLLHK<br>LEKGTNRKSKKPPAFAKKGMKVIAVLETEAPVCVETYQDYPQLGRFTLRDQGTTIAIGKIVKIAEGAPGSAG<br>SAAGSGMVSKGEELFTGVVPILVELDGDVNGHKFSVSGEGEGDATYGKLTLKFICTTGKLPVPWPTLVTTLT<br>YGVQCFSRYPDHMKQHDFFKSAMPEGYVQERTIFFKDDGNYKTRAEVKFEGDTLVNRIELKGIDFKEDGNI<br>LGHKLEYNYNSHNVYIMADKQKNGIKVNFKIRHNIEDGSVQLADHYQQNTPIGDGPVLLPDNHYLSTQSK<br>LSKDPNEKRDHMVLLEFVTAAGITLGMDELYK
PhIF	MARTPSRSSIGSLRSPHTHKAILTSTIEILKECGYSGLSIESVARRAGASKPTIYRWWTNKAALIAEVYENESE<br>QVRKFPDLGSFKADLDFLLRNLWKVWRETICGEAFRCVIAEAQLDPATLTQLKDQFMERRREMPKKLVEN<br>AISNGELPKDTNRELLLDMIFGFCWYRLLTEQLTVEQDIEEFTFLLINGVCPGTQREFPPKKKRKVSTAPPTD<br>VSLGDELHLDGEDVAMAHADALDDFDLDMLGDGDSPGPGFTPHDSAPYGALDMADFEFEQMFTDALG<br>IDEYGG

The underlined part indicates the amino acid sequence of GFP.

Table S8. *Saccharomyces cerevisiae – Escherichia coli* shuttle plasmids used in this study.

Plasmid name	Plasmid type	Yeast marker	Promoter	Expression cassette	Origin
P0-Venus	*CEN*	*LEU2*	Synthetic promoter-P0	*Venus*	(36)
P1-Venus	*CEN*	*LEU2*	Synthetic promoter-P1	*Venus*	(36)
P2-Venus	*CEN*	*LEU2*	Synthetic promoter-P2	*Venus*	(36)
P4-Venus	*CEN*	*LEU2*	Synthetic promoter-P4	*Venus*	(36)
P8-Venus	*CEN*	*LEU2*	Synthetic promoter-P8	*Venus*	(36)
P0-Sup35-GFP	*CEN*	*LEU2*	Synthetic promoter-P0	*Sup35-GFP*	This study
P1-Sup35-GFP	*CEN*	*LEU2*	Synthetic promoter-P1	*Sup35-GFP*	This study
P2-Sup35-GFP	*CEN*	*LEU2*	Synthetic promoter-P2	*Sup35-GFP*	This study
P4-Sup35-GFP	*CEN*	*LEU2*	Synthetic promoter-P4	*Sup35-GFP*	This study
P8-Sup35-GFP	*CEN*	*LEU2*	Synthetic promoter-P8	*Sup35-GFP*	This study
P413-Gal-Sup35-GFP	*CEN*	*HIS3*	P_{GAL1}	*Sup35-GFP*	This study
pAG415Sup35-Sup35WT-GFP	*CEN*	*LEU2*	P_{Sup35}	*Sup35-GFP*	(33)
P_{TDH3}-PhIF	*CEN*	*URA3*	P_{TDH3}	*PhIF*	(36)

Table S9. Sequence of synthetic promoters of SynPro plasmids used in this study.

<table>
<tr><th rowspan="2">Plasmid</th><th colspan="2">Synthetic promoter</th><th>Origin</th></tr>
<tr><th>ENO1 Core promoter (ENO1cp)</th><th>Binding site (engineered upstream of ENO1cp)</th><th></th></tr>
<tr><td>P0</td><td rowspan="5">CTCCCCGGAAACTGTGGCCTTTTCTGGCACACATGATCTCCACGATTTCAACATATAAATAGCTTTATGATACGAAACGTACCGTATCGTTAAGGTTAAGCCATGATACGAAACGTACCGTATCGTTAAGGTTCTTGTAATCCCTTATTCCTTCTAGCTATTTTTCATAAAAAAACCAAGCAACTGCTTATCAACACACAAACAC</td><td>-</td><td>(36)</td></tr>
<tr><td>P1</td><td>ACCTTAACGATACGGTACGTTTCGTATCAT</td><td>(36)</td></tr>
<tr><td>P2</td><td>ACCTTAACGATACGGTACGTTTCGTATCATTCACATCCTAGGTCTACCTTAACGATACGGTACGTTTCGTATCAT</td><td>(36)</td></tr>
<tr><td>P4</td><td>ACCTTAACGATACGGTACGTTTCGTATCATATTGACAAGCTTCAGACCTTAACGATACGGTACGTTTCGTATCATGGCTTATCTAGAGTCACCTTAACGATACGGTACGTTTCGTATCATTCACATCCTAGGTCTACCTTAACGATACGGTACGTTTCGTATCAT</td><td>(36)</td></tr>
<tr><td>P8</td><td>ACCTTAACGATACGGTACGTTTCGTATCATATTGACAAGCTTCAGACCTTAACGATACGGTACGTTTCGTATCATGGCTTATCTAGAGTCACCTTAACGATACGGTACGTTTCGTATCATCCTAGGTCTACCTTAACGATACGGTACGTTTCGTATCATACTAGCACCTTAACGATACGGTACGTTTCGTATCATATTGACAAGCTTCAGACCTTAACGATACGGTACGTTTCGTATCATGGCTTATCTAGAGTCACCTTAACGATACGGTACGTTTCGTATCATTCACATCCTAGGTCTACCTTAACGATACGGTACGTTTCGTATCAT</td><td>(36)</td></tr>
</table>

The underlined part indicates the repeating sequence of a binding site.
